# Ferrodoxin 1 (FDX1) drives paclitaxel resistance in ovarian cancer via copper metabolism and ULK1/ATG13-mediated autophagy: overcome by pH/ROS-responsive PPD/PDP@si-FDX1 nanomicelles

**DOI:** 10.1186/s13046-025-03589-z

**Published:** 2026-04-23

**Authors:** Yangmei Gong, Zhizhi Deng, Jie Wu, Yi Hu

**Affiliations:** The First Affiliated Hospital, Center for a combination of Obstetrics and Gynecology & Reproductive medicine, Henyang Medical School, University of South China, No. 69, Chuanshan Avenue, Hengyang, 421001 Hunan Province China

**Keywords:** Ovarian cancer, Paclitaxel resistance, PH/Reactive oxygen Species-Responsive nanomicelles, Ferredoxin 1, Unc-51 like autophagy activating kinase 1, Autophagy related 13, Autophagy, Cuproptosis

## Abstract

**Background:**

Ovarian cancer (OC) is often characterized by poor prognosis due to paclitaxel (TAX) resistance, with ferredoxin 1 (FDX1) emerging as a key mediator of copper metabolism. The present study aimed to elucidate the role of FDX1 in TAX resistance and to evaluate the efficacy of pH/reactive oxygen species (ROS)-responsive nanomicelles (PPD/PDP@si-FDX1) in reversing this resistance.

**Methods:**

TAX-resistant A2780 and SKOV3 cells were established by gradient exposure, and FDX1 expression was assessed using Western blotting. FDX1 was either overexpressed or silenced, and resistance was evaluated using CCK-8, clonogenic, scratch, and Transwell assays. Autophagic activity was examined through Western blotting, immunofluorescence, and transmission electron microscopy. Mechanistic validation involved the ULK1 activator BL-918 and the copper chelator TTM. PPD/PDP@si-FDX1 nanomicelles were prepared via self-assembly, with structural and responsive characteristics analyzed by transmission electron microscopy (TEM), dynamic light scattering (DLS), and drug release profiling. Tumor penetration and in vivo antitumor efficacy were examined using multicellular spheroids and subcutaneous xenograft models.

**Results:**

FDX1 overexpression elevated intracellular copper, activated the ULK1/ATG13 autophagic axis, and enhanced TAX resistance; silencing FDX1 reversed these effects. Copper chelators or ULK1 inhibition phenocopied FDX1 silencing. PPD/PDP@si-FDX1 demonstrated pH/ROS-responsive tumor accumulation and enhanced si-FDX1 delivery. In vivo, it significantly suppressed tumor growth and restored TAX sensitivity, outperforming free si-FDX1.

**Conclusion:**

FDX1 drives TAX resistance via copper-dependent ULK1/ATG13 activation; PPD/PDP@si-FDX1 nanomicelles effectively reverse resistance, offering a promising strategy for OC therapy.

**Graphical Abstract:**

Graphic abstract. The molecular mechanism of PPD/PDP@si-FDX1 exhibits antitumor effects

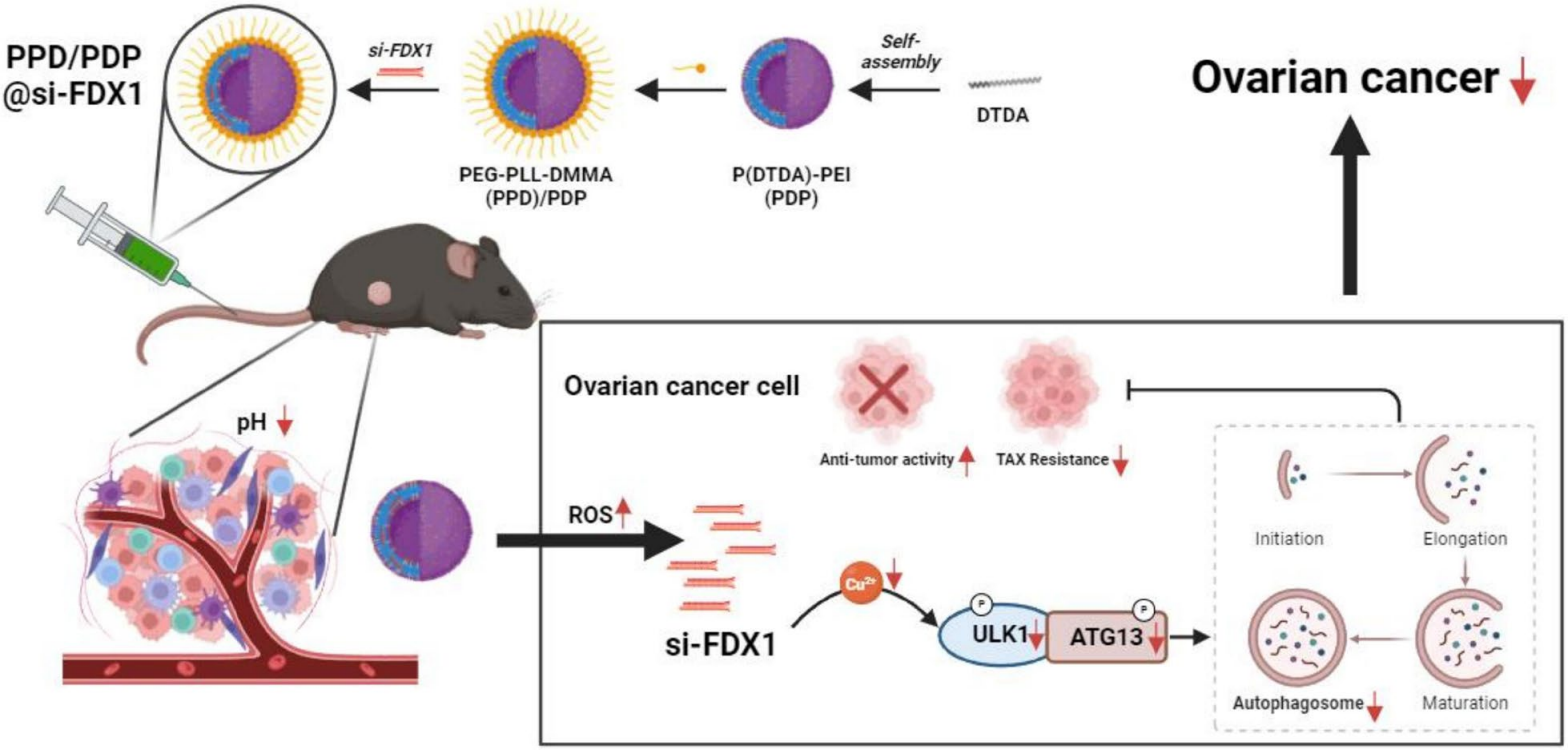

**Supplementary Information:**

The online version contains supplementary material available at 10.1186/s13046-025-03589-z.

## Introduction

Ovarian cancer (OC) is one of the most common malignant tumors in the female reproductive system, with thousands of new cases diagnosed worldwide each year. It accounts for approximately 3.7% of all female cancers and 4.7% of cancer-related deaths [1]. Despite recent advancements in early screening and diagnostic technologies, OC often remains undetected in its early stages due to subtle symptoms, leading to late-stage diagnoses in most patients and poor prognosis [2, 3]. The primary treatment for OC involves surgical resection and chemotherapy, with Paclitaxel (TAX) being the first-line chemotherapeutic drug [4, 5]. TAX exerts its anti-tumor effects by stabilizing microtubule protein polymerization, inhibiting tumor cell division and proliferation [6]. However, resistance to TAX is a major clinical challenge, resulting in poor treatment outcomes and worsened prognosis [7]. TAX resistance has emerged as a significant obstacle in OC treatment, underscoring the importance of elucidating its mechanisms and developing new approaches to enhance treatment outcomes [3, 8, 9].

The mechanisms underlying TAX resistance are complex, involving various cellular and molecular changes [8]. Autophagy is an intracellular degradation process that maintains cellular stability by degrading damaged organelles and proteins [10]. Under certain conditions, autophagy is activated to counter cell stress, enhancing cell survival and potentially contributing to drug resistance in tumor cells [11–13]. Ferredoxin 1 (FDX1), a key protein in mitochondrial iron-sulfur cluster synthesis, has recently been linked to copper metabolism and autophagy [14]. Studies suggest that FDX1 modulates autophagic activity by regulating intracellular copper levels, thereby influencing drug resistance in tumor cells [15]. In OC, FDX1 expression is closely associated with TAX resistance, indicating that FDX1 may be a potential target to overcome TAX resistance [16–18].

With advancements in nanotechnology, nanomedicine delivery systems have gained significant attention in cancer treatment. These systems target cancer biomarkers and signaling pathways to improve treatment efficacy and reduce drug resistance [19]. By encapsulating drugs or gene vectors in nanoparticles, they enhance drug stability, targeting specificity, and bioavailability, while minimizing adverse effects on normal tissues [20–22]. Recently, pH and reactive oxygen species (ROS)-responsive nanomicelles have emerged as a crucial strategy in nanodelivery systems [23, 24]. Copper, an essential trace element, plays a crucial role in tumor drug resistance due to its homeostatic imbalance. “Cuproptosis” is a copper-induced cell death process in which mitochondrial Cu(I) binds to TCA cycle lipoylated proteins, such as DLAT and DLST, causing protein aggregation and loss of Fe–S clusters. FDX1 regulates susceptibility to cuproptosis through the LIAS-mediated lipoylation axis. Additionally, copper acts as a signaling molecule to activate ULK1/2, initiating autophagy and forming a “Cu → ULK1 → autophagy” survival pathway. Under chemotherapeutic stress, the “copper–lipoylation–death” and “copper–ULK1–autophagy–survival” pathways compete and jointly affect drug resistance. The acidic tumor microenvironment (TME) and elevated ROS levels trigger drug release from nanomicelles, enabling precise drug delivery in tumor tissues [25]. The strategy of pH/ROS-responsive nanomicelles loaded with small interfering RNA (siRNA) targeting FDX1 (PPD/PDP@si-FDX1) shows promise in reversing TAX resistance in OC by suppressing FDX1 expression and modulating autophagic pathways. This approach offers high targeting specificity, low toxicity, and controlled drug release, making it an effective option for combating highly resistant tumor cells.

Significantly, this research introduces the novel concept that FDX1 regulates autophagy through the copper metabolism pathway, affecting TAX resistance in OC, addressing a research gap in the field. The study also presents a pH/ROS-responsive nanomicelles system as an innovative strategy to overcome tumor resistance. By precisely controlling drug release, this nanodelivery system enhances drug penetration and anti-tumor activity, showing considerable potential for clinical applications. Moreover, the nanomicelles technology is not limited to OC and may be applicable to other drug-resistant tumors. The findings provide a new approach to overcoming TAX resistance and offer valuable insights for the future development and application of anticancer therapies.

## Materials and methods

### Public data download and analysis

Transcriptomic profiles of 427 °C cases were downloaded from The Cancer Genome Atlas (TCGA) database (https://portal.gdc.cancer.gov/). Due to the absence of normal tissue samples in TCGA, 88 normal ovarian tissue samples were retrieved from the Genotype-Tissue Expression (GTEx) database (https://www.gtexportal.org/home/datasets) to compare FDX1 mRNA expression between OC and normal tissues. Additionally, proteinomic data of FDX1 were downloaded from The National Cancer Institute’s Clinical Proteomic Tumor Analysis Consortium (CPTAC) (PDC000110), comprising 25 normal and 100 tumor tissue samples. Ethical approval was not required as the data were sourced from public databases.

### Establishment of Paclitaxel-Resistant cell lines (A2780-TR and SKOV3-TR)

Cell origin and culture conditions: The parental OC cell lines A2780 and SKOV3 were cultured under standard conditions (37 °C, 5% CO₂) in complete medium supplemented with 10% fetal bovine serum (FBS). TAX was dissolved in DMSO to prepare a stock solution, stored at − 20 °C in the dark. The final concentration of DMSO in all treatments was maintained at ≤ 0.2%.

Stepwise induction of TAX resistance: The resistant cell lines were established through long-term stepwise exposure, as previously described [26]. Cells were treated with increasing concentrations of TAX (2, 5, 10, 20, and 50 nM). Each step involved the following cycle: (1) treatment with TAX for 24 h; (2) replacement with drug-free complete medium for 48–72 h to allow surviving cells to recover and proliferate; (3) passage of cells for 2–3 stable generations at each concentration (cell viability >80%, stable proliferation curve) before escalating to the next dose.

Cells that could grow stably and continuously for at least five passages at 10 nM TAX were designated as resistant sublines, A2780-TR and SKOV3-TR. To maintain the resistant phenotype, low-dose TAX (e.g., 5 nM) was added to the maintenance medium during routine culture.

Cell Viability Assay: Parental and resistant cells were seeded in 96-well plates (approximately 5 × 10³ cells/well, adjusted according to proliferation rate). After attachment, cells were treated with varying concentrations of TAX (0, 2, 5, 10, 20, 50 nM) for 48 h. Following treatment, CCK-8 reagent was added and incubated for 1–2 h. Absorbance was recorded at 450 nm, and the values were normalized to the 0 nM control to determine relative cell viability. Each concentration was tested in at least three technical replicates, and all experiments were repeated independently at least three times. Drug sensitivity and IC₅₀ determination results are shown in Figure S1 C.

### In *Vitro* Cell Cultures

Human OC cell lines A2780 (ml095891, Shanghai Enzyme-linked Biotechnology, China) and SKOV3 (ml097240, Shanghai Enzyme-linked Biotechnology, China), along with the drug-resistant sublines A2780-TR and SKOV3-TR established in our laboratory, were cultured in RPMI-1640 medium (R4130, Sigma-Aldrich, USA) enriched with 10% FBS (F8687, Sigma-Aldrich, USA). Cultures were incubated at 37 °C in a humidified atmosphere containing 5% CO₂, and subcultured once reaching approximately 80–90% confluence.

The HEK293T cell line (CRL-3216, ATCC) was cultured in DMEM medium (11965092, Gibco, USA) containing 10% FBS, 10 µg/mL streptomycin, and 100 U/mL penicillin. Cells were maintained under the same incubation conditions (37 °C, 5% CO₂) and passaged at 80–90% confluence.

### Cell transfection and grouping

Three small interfering RNA (siRNA) sequences targeting FDX1 were designed based on the GenBank reference sequence, while a scrambled siRNA served as the negative control (si-NC). The primer sequences are listed in Table S1, and the specific siRNA sequences were synthesized by GenePharma. Transfection of siRNA into A2780-TR and SKOV3-TR cells was performed using Lipofectamine 2000 (11668030, Thermo Fisher, USA) when the cell confluence reached 80%−90%. Transfection was carried out according to the product manual, and cells were harvested 24–48 h post-transfection for subsequent experiments.

For FDX1 overexpression, the coding sequence was cloned into the pLV-EF1α-MCS-PGK-Puro vector, in which EF1α drives FDX1 expression and the PGK promoter regulates puromycin resistance for stable selection. High-titer lentivirus was generated by Genechem (Shanghai, China) and used to infect A2780 and SKOV3 cells. After 24 h, the infection medium was replaced, and cultures were maintained for an additional 48 h before selection with 1 µg/mL puromycin (Sigma-Aldrich, USA) to establish stable FDX1-overexpressing lines.

Cell grouping was as follows: For A2780 and SKOV3 cells, (1) overexpression-negative control (oe-NC) group, oe-FDX1 group; (2) DMSO group, Bafilomycin A1 (BafA1) group; (3) oe-FDX1 + DMSO group, oe-FDX1 + 3-MA group, oe-FDX1 + TTM group. For A2780-TR and SKOV3-TR cells, (1) si-NC group, small interfering RNA targeting FDX1 (si-FDX1) group; (2) si-FDX1 + DMSO group, si-FDX1 + BL-918 group. DMSO treatment was uniform across groups with cells treated with 10 nM BafA1 (Med Chem Express, Catalog no. HY-100558) for 24 h, 5 mM 3-MA (HY-19312, Med Chem Express, China) for 24 h, 10 µM Unc-51 Like Autophagy Activating Kinase 1 (ULK1) inhibitor MRT68921 (HY-100006, Med Chem Express, China) for 24 h, 5 µM ULK1 activator BL-918 (HY-124729, Med Chem Express, China) for 24 h, and 30 µM cuproptosis inhibitor TTM (HY-128530, Med Chem Express, China) for 24 h. (3) si-FDX1 group: Cells were not treated with BafA1 or BL-918. si-FDX1 + BafA1 group: Cells were treated with 10 nM BafA1 for 24 h prior to sample collection. si-FDX1 + BL-918 group: Cells were treated with 10 µM BL-918 for 24 h without BafA1. si-FDX1 + BL-918 + BafA1 group: Cells were first treated with 10 µM BL-918 for 24 h, followed by an additional 24-hour treatment with 10 nM BafA1.

### RT-qPCR

Total RNA was isolated using the Trizol Reagent Kit (T9424, Sigma-Aldrich, USA), and its purity and concentration were determined with a NanoDrop spectrophotometer (ND-1000, Thermo Fisher Scientific, USA). Complementary DNA (cDNA) was synthesized using the PrimeScript™ RT-qPCR Kit (RR086A, TaKaRa, Mountain View, CA, USA). Quantitative PCR was performed with SYBR Green Master Mix (4364344, Applied Biosystems, USA) on the ABI PRISM 7500 Detection System. GAPDH served as the internal reference for normalization. Primer sequences used for amplification were designed by General Biotech (Shanghai, China) and are listed in Table S2. Relative gene expression was calculated using the 2^−ΔΔCt^ method.

### Western blot (WB)

Proteins were extracted from tissues or cultured cells using RIPA lysis buffer supplemented with protease inhibitors (P0013B, Beyotime, Shanghai, China). Protein concentrations were measured by the BCA Protein Assay Kit (P0012, Beyotime, Shanghai, China). Equal amounts of protein were separated on 10% SDS-PAGE gels and transferred onto PVDF membranes. After blocking with 5% bovine serum albumin (BSA) for 2 h at room temperature, membranes were incubated with specific primary antibodies (listed in Table S3) for 1 h. Following washing, HRP-conjugated goat anti-rabbit IgG (ab6721, 1:2000, Abcam, UK) was applied for 1 h at ambient temperature. Immunoreactive bands were visualized using the Pierce™ ECL substrate (32209, Thermo Fisher Scientific, USA) and captured with a Bio-Rad imaging system. Band intensity was quantified using ImageJ software, with GAPDH serving as the internal loading control. All experiments were independently repeated three times.

### In *Vitro* Kinase Activity Assay

In this assay, 100 ng of recombinant GST-Autophagy Related 13 (ATG13) (600-401-C49, Rockland, USA) was incubated with or without 1 µg of recombinant GST-ULK1 (PV4814, Thermo Fisher Scientific, USA), HA-ULK1WT, or HA-ULK1 CBM in a kinase buffer (25 mM Tris-HCl [pH 7.5], 20 mM MgCl2, 2 mM dithiothreitol [DTT], 25 mM β-glycerophosphate [β-GP], 0.5 mM Na_3_VO_4_, 120 µM ATP) for 30 min at 30 °C.

To assess the impact of ULK1 on ATG13 phosphorylation, proteins were separated by SDS-PAGE following the reaction and analyzed by WB using anti-phospho-ATG13 (P-ATG13) antibodies. Total ATG13 protein levels were confirmed using anti-total-ATG13 (T-ATG13) antibodies to ensure consistent protein quantity. For further analysis, varying concentrations of CuCl2 (451657, Sigma-Aldrich, USA) were gradually added to the reaction mixture (0 to 10 µM) to quantitatively assess ATG13 phosphorylation levels via WB. Additionally, the effect of the copper chelator TTM (0 to 50 µM) on ATG13 phosphorylation was examined to evaluate the role of copper ions in ULK1-mediated phosphorylation. Finally, the impact of a 10 µM ULK1/2 kinase inhibitor (BL-918) on ATG13 phosphorylation was analyzed by WB to assess its inhibitory effect.

### Immunocomplex kinase assay

To evaluate ULK1 kinase activity, an immunocomplex kinase assay was performed following immunoprecipitation (IP) of ULK1 protein. HEK293T cells were washed with cold PBS and lysed in cold NP-40 lysis buffer containing 1X EDTA-free Halt™ Protease and Phosphatase Inhibitor Cocktail (78442, Thermo Fisher Scientific, USA). Protein concentrations were determined using the BCA Protein Assay Kit, with BSA as the standard. A total of 1.5 mg of protein lysate was used to immunoprecipitate endogenous or exogenous ULK1 with rabbit anti-ULK1 antibody (8054, 1:50, Cell Signaling, China), which was incubated overnight at 4 °C. After incubation, 20 µL of Protein A Agarose Beads (9863, Cell Signaling, China) was added, and the mixture was incubated for an additional 3 h. The beads were washed three times with NP-40 buffer and once with PBS. The beads were then resuspended in 90 µL of kinase buffer, and each reaction was supplemented with 100–500 ng of recombinant GST-ATG13, followed by incubation at 30 °C for 30 min.

### Copper ion detection

Copper concentrations in tissue or cell samples were measured using a Copper Assay Kit (MAK127, Sigma-Aldrich, USA). Absorbance was detected at 359 nm with an Infinite 200 microplate reader (Tecan, Beijing, China), and concentrations were calculated based on a standard calibration curve.

### Cell viability assay using cell counting Kit-8 (CCK-8)

OC cells from various groups were treated with various concentrations of TAX (2, 5, 10, 20, 50 nM) for 48 h, followed by assessment of cell viability using the CCK-8 assay. Cells were digested, resuspended, and adjusted to a concentration of 1 × 10⁵ cells/mL. A 100 µL suspension of cells was seeded into each well of a 96-well plate and cultured under standard conditions until adherence. After drug treatment, cells were incubated overnight. Viability was assessed on days 1 and 4 post-treatment (C0041, Beyotime, Shanghai, China). For each measurement, 10 µL of CCK-8 solution was added and incubated for 4 h. Absorbance was recorded at 450 nm using a spectrophotometer, and cell survival rates were calculated.

### Clonogenic assay to assess cell clonogenicity

Cells in the logarithmic growth phase were collected, digested, and resuspended as single-cell suspensions with viability exceeding 95%. After counting, the suspensions were diluted to the desired concentration, and approximately 100 cells in 5 mL medium were seeded into each 60 mm culture dish. The dishes were gently shaken to ensure even distribution of cells and incubated at 37 °C with 5% CO₂ for 2–3 weeks. Once visible colonies formed, the medium was discarded, and the dishes were washed twice with PBS and air-dried. Colonies were fixed with methanol for 15 min, air-dried, stained with Giemsa for 10 min, and gently rinsed with running water. After air-drying, colonies with more than 10 cells were counted by eye or under a microscope at low magnification. Clonogenic efficiency was calculated using the formula: Clonogenic Efficiency = (Number of Colonies/Number of Cells Seeded) × 100%.

### Scratch assay

A scratch assay was performed to assess cell migration. Uniform horizontal lines, spaced 1 cm apart, were drawn on the back of a 6-well plate. Approximately 5 × 10⁵ cells were seeded per well and cultured until 100% confluence. A pipette tip was used to create a vertical scratch along the marked lines. After scratching, the wells were washed three times with sterile PBS to remove non-adherent cells, ensuring the scratch gap remained visible. The cells were then incubated in fresh serum-free medium at 37 °C and 5% CO₂. After 24 h, the width of the scratch was observed under a microscope, photographed, and migration rates were analyzed using ImageJ software.

### Transwell assay

Cell invasion was assessed using Transwell chambers with 8 μm pores (3391, Corning, USA). Matrigel (354277, BD Biosciences, USA) was applied to each well. Cells from each group (2 × 10⁵ cells) were suspended in serum-free medium and added to the upper chamber. After incubation at 37 °C for 24 h, the migrated cells were stained with 0.5% crystal violet for 20 min at ambient temperature. The stained cells were imaged under a Nikon Eclipse Ci microscope (Nikon, Tokyo, Japan), and five random visual fields were selected for counting. Mean values were calculated to quantify invasion ability.

### Proteomics sample Preparation and measurement

Cell lysates from the si-NC (*n* = 3) and si-FDX1 (*n* = 3) groups of A2780-TR cells were collected in 5 cm³ centrifuge tubes. Cells were lysed on ice using an ultrasonic cell disruptor (SCIENTZ-IID, Scientz, Ningbo, China). Lysis was performed in phenol extraction buffer containing 10 mM DTT, 1% protease inhibitor mixture, and 2 mM EDTA (Solarbio, Beijing, China). The sonication process was repeated eight times to ensure complete cell disruption. Equal volumes of Tris-saturated phenol (pH 8.0, BIOFOUNT, Beijing, China) were added, followed by vortexing for 4 min. Samples were centrifuged at 5000×g for 10 min at 4 °C, and the phenolic upper phase was transferred to new tubes. To precipitate proteins, 0.1 M ammonium sulfate-saturated methanol (101217 and 106035, Merck, USA) was added to the phenol phase at a 1:5 volume ratio and incubated overnight at 4 °C. After centrifugation for 10 min, the supernatant was discarded. The protein pellet was washed three times with cold methanol and acetone, then redissolved in 8 M urea (U8020, Solarbio, Beijing, China). Protein concentrations were determined using a BCA Protein Assay Kit (P0012, Beyotime, Shanghai, China).

### Proteolytic Digestion, peptide Labeling, and Nano-LC-MS/MS analysis

For proteolytic digestion, 50 µg of total protein from each sample was processed. Protein solutions were first reduced with 5 mM DTT at 56 °C for 30 min, followed by alkylation with 11 mM iodoacetamide at ambient temperature for 15 min in the dark. The urea concentration was then diluted to below 2 M, and sequencing-grade trypsin (25200056, Thermo Fisher Scientific, USA) was added at an enzyme-to-protein ratio of 1:50 (w/w) for overnight incubation at 37 °C. A second digestion step was performed for 4 h at a 1:100 ratio to ensure complete cleavage.

Resulting peptides were desalted on HyperSep™ C18 columns (60108-302, Thermo Fisher Scientific, USA) and vacuum-dried. The peptides were redissolved in 0.5 M TEAB (90114, Thermo Fisher Scientific, USA) and labeled using the TMT kit (90064CH, Thermo Fisher Scientific, USA). A unit of TMT reagent was thawed and reconstituted in acetonitrile (113212, Merck, USA). The peptide mixtures were incubated at ambient temperature for 2 h, then desalted and vacuum-dried. The combined samples were fractionated into 15 components using the Pierce™ High-pH Reversed-Phase Peptide Fractionation Kit (84868, Thermo Fisher Scientific, USA), dried, and reconstituted in 0.1% formic acid (159002, Merck, USA) for LC–MS/MS analysis.

For LC-MS/MS, 2 µg of each peptide sample was separated using the Easy nLC 1200 nano-UPLC system (Thermo Fisher Scientific, USA). Peptides were first loaded onto a Trap C18 column (100 μm × 20 mm, 5 μm) and subsequently resolved on a C18 analytical column (75 μm × 150 mm, 3 μm) at a constant flow rate of 300 nL/min. The mobile phase consisted of 0.1% formic acid in water (solvent A) and 0.1% formic acid in 95% acetonitrile (solvent B). The gradient elution program was as follows: 0–2 min, 2–8% B; 2–71 min, 8–28% B; 71–79 min, 28–40% B; 79–81 min, 40–100% B; and 81–90 min, 100% B. Mass spectrometric detection was performed on a Q-Exactive HFX instrument (Thermo Fisher Scientific, USA) operated in positive electrospray ionization mode (ESI+, 2.1 kV) for 60 min. The full MS scan was acquired over an m/z range of 350–1200 at a resolution of 60,000 (m/z 200), with an AGC target of 3 × 10⁶ and a maximum injection time of 30 ms. MS/MS spectra were collected at a resolution of 15,000 (m/z 200) using higher-energy collisional dissociation (HCD) with a normalized collision energy of 32, an AGC target of 1 × 10⁶, a maximum injection time of 25 ms, and an isolation window of 2.0 Th.

### Proteomics data analysis

Raw LC–MS/MS data were analyzed using MaxQuant software (version 1.5.2.8) for peptide identification and quantitative protein profiling. Database searches were performed against the UniProt 14.1 (2009) Gossypium hirsutum proteome combined with a reverse decoy database to estimate false discovery rates (FDR). Trypsin/P was set as the cleavage enzyme, allowing up to two missed cleavages. The precursor mass tolerances were defined as 20 ppm for the initial search and 5 ppm for the main search, while fragment ion tolerance was set to 0.02 Da. Protein and peptide identifications were filtered at a false discovery rate ≤ 1%, based on score distribution. Differential expression analysis between groups was performed with the limma package in R, applying |log₂FC| >1 and *p* < 0.05 as significance thresholds. Enrichment analysis of differentially expressed proteins (DEPs) was conducted using the ClusterProfiler package in R, with *p* < 0.05 as the cutoff for Gene Ontology (GO) and KEGG pathway enrichment.

### Immunofluorescence staining

Confocal immunofluorescence imaging was performed on tumor tissues and cells. Tissue sections were first treated with 3% hydrogen peroxide to quench endogenous peroxidase activity, followed by blocking with serum or 1% BSA to prevent nonspecific binding. Cells were fixed with 4% paraformaldehyde (Yeasen Biotechnology, China), permeabilized using 0.2% Triton X-100 (Beyotime, Shanghai, China), and blocked in PBS containing 1% BSA.

Primary antibodies against FDX1 (ab108257, Abcam, UK) and LC3B (ab232940, Abcam, UK) were diluted at 1:100 and incubated overnight at 4 °C. The following day, secondary antibodies, either Alexa Fluor 594 conjugated (ab150080, Abcam, UK) diluted 1:500 or Alexa Fluor™ 488 conjugated goat anti-rabbit IgG (A-11008, Invitrogen, USA) diluted 1:1000, were applied and incubated for 1 h at ambient temperature in the dark. After incubation, tissues and cells were washed three times with PBS for 5 min each. Nuclei were stained with DAPI (ab104139, 1:1000, Abcam, UK).

For mitochondrial staining, cells were incubated with 400 nM MitoTracker Deep Red (M22426, Invitrogen, USA) at 37 °C for 30 min. Finally, samples were mounted using ProLong™ Gold antifade mountant (P36930, Invitrogen, USA) and imaged with an LSM 700 confocal microscope (Carl Zeiss, Germany) to visualize FDX1 and LC3B localization.

### Mitochondrial membrane potential (MMP) assay

MMP was measured using the JC-1 MMP Assay Kit (Yeasen Biotechnology, Shanghai, China). Cells were seeded in 6-well plates, and 1 mL of JC-1 staining solution was added to each well and mixed thoroughly. After incubation at 37 °C for 20 min, a positive control was prepared by treating cells with 50 µM carbonyl cyanide 3-chlorophenylhydrazone (CCCP, diluted 1:1000 from a 50 mM stock provided in the kit) for an additional 20 min. Following incubation, the staining solution was removed, and the cells were washed twice with JC-1 buffer. Two milliliters of culture medium (including serum and phenol red if required) were then added. Cells were examined using a fluorescence or confocal laser microscope. JC-1 monomers were detected using excitation at 490 nm and emission at 530 nm.

### Transmission electron microscopy (TEM) for mitochondrial morphology

Cells were fixed overnight at 4 °C in 2.5% glutaraldehyde, followed by fixation in 1% osmium tetroxide for 1–2 h. Samples were dehydrated through a graded ethanol series (50%, 70%, 80%, 90%, and 95%) and then treated with pure acetone. After dehydration, samples were infiltrated overnight with pure embedding resin and polymerized at 70 °C overnight. Ultrathin Sects. (70–90 nm) were cut using a Reichert ultramicrotome and stained for 15 min each with lead citrate and 50% ethanol-saturated uranyl acetate. The stained sections were examined under a TEM to assess mitochondrial morphology.

### Preparation of nanomicelles

N⁶-Benzoxycarbonyl-L-lysine (25 mmol) was dissolved in tetrahydrofuran (THF) and stirred at 50 °C for 10 min, followed by the gradual addition of triphosgene (14 mmol). The reaction mixture was stirred for 5 h, poured into n-hexane, and stored at − 20 °C for 24 h. The precipitate was filtered, washed with n-hexane, and vacuum-dried to yield N6-Benzoxycarbonyl-L-lysine N-carboxyanhydride (Lys-NCA). Using mPEG-NH₂ as an initiator, PEG-PLLZ was synthesized through ring-opening polymerization of Lys-NCA. The benzoxycarbonyl groups of PEG-PLLZ were then removed to obtain PEG-PLL. The pH was adjusted to 8.5 with NaOH, followed by the addition of dimethylmaleic anhydride (DMMA) and stirring for 12 h. After dialysis and lyophilization, PEG-PLL-DMMA (PPD) was obtained.

For the synthesis of P(DTDA), 2.45 mmol of 2-mercaptoethanol and 4.9 mmol of acetone were mixed and stirred at ambient temperature for 6 h. After crystallization in an ice bath, the crystals were washed with n-hexane and cold water, and the product 5,5-dimethyl-4,6-dithionane diethanol (DTDA) was vacuum-dried. Under a nitrogen atmosphere, DTDA (1.5 mmol) and triphosgene (8 mmol) were dissolved in dichloromethane (DCM) and stirred for 3 h, then left to react overnight at ambient temperature. The solvent was removed, and the residue was redissolved in DCM, followed by the addition of pyridine (9 mmol) and stirring for 24 h. The concentrated solution was cooled, centrifuged, and washed with methanol to yield a yellow solid, P(DTDA). To prepare P(DTDA)-PEI (PDP), P(DTDA) (1 mmol), dicyclohexylcarbodiimide (DCC) (1.2 mmol), and N-hydroxysuccinimide (NHS) (1.2 mmol, J&K Scientific, Beijing, China) were dissolved in 30 mL DMSO and stirred at ambient temperature for 1.5 h. Polyethyleneimine (PEI, Mw 1800, 0.5 mmol) was added, and the mixture was stirred for 24 h. The resulting solution was dialyzed (MWCO 2.0 kDa) for 4 days and lyophilized to yield the copolymer PDP.

Preparation of PPD/PDP@si-FDX1: PDP (14.8 mg) was dissolved in 2 mL DMSO and stirred at ambient temperature for 1 h. The solution was then added dropwise into 5 mL of double-distilled water, adjusted to pH 7.4, and dialyzed (MWCO 3.5 kDa) for 24 h. PPD (33.2 mg) was dissolved in 33.2 mL of water (pH 8.0), stirred for 30 min at ambient temperature, and incubated overnight before lyophilization. PPD/PDP was mixed with siRNA at various mass ratios (1:1 to 15:1) to form PPD/PDP@si-FDX1 complexes. Complex formation was verified by loading the mixtures onto 1% agarose gels and performing electrophoresis in Tris-Acetate-EDTA (TAE) buffer at 100 V. After electrophoresis, gels were stained with ethidium bromide, and siRNA migration was visualized under UV light.

### Characterization of nanomicelles

The morphology of nanomicelles under different conditions (pH 6.8, pH 7.4, and after H₂O₂ treatment) was examined using a TEM (JEM-1011, JEOL, Tokyo, Japan) operated at 200 kV. The average particle size distribution, zeta potential, and polydispersity index (PDI) were measured using a Malvern Zetasizer Nano ZS90 particle analyzer (Malvern Instruments, UK). High-performance liquid chromatography (HPLC) was performed on a C18 column (Bioband HP-120, 4.6 mm × 150 mm, 5 μm) using a linear gradient of methanol and deionized water as the mobile phase, with a flow rate of 0.8 mL/min at 29 °C.

### Protein adsorption experiment

BSA was used as a model protein to evaluate micelle adsorption capacity under different pH conditions. PDP (1 mg/mL) was incubated with 2 mg/mL BSA in PBS at pH 7.4 or 6.0 at 37 °C for 12 h. Each sample was then centrifuged to separate micelles with adsorbed protein. The residual BSA concentration in the supernatant was measured using a UV-visible spectrophotometer (Lambda 900, PerkinElmer Instruments, USA), and the amount of protein adsorbed on the micelles was calculated from the difference in BSA concentration.

### In *Vitro *Drug Release Experiment

Hoechst 33,342-labeled si-FDX1 (14533, Sigma-Aldrich, USA) was loaded into PPD/PDP micelles (3 mg) and suspended in 1 mL of PBS (pH 7.4) containing different concentrations of H₂O₂ (0, 0.1 mM, and 1 mM). The suspension was placed in a dialysis bag (MWCO 3.5 kDa; Sigma-Aldrich, USA) and immersed in 29 mL of PBS (pH 7.4) under dark conditions. At designated time intervals, 0.6 mL of the external solution was collected, and the release of Hoechst-si-FDX1 was measured using a fluorescence spectrophotometer at 460 nm. An equal volume of fresh PBS was added after each sampling to maintain constant volume and buffer composition.

### Cultivation of A2780 MCSs and penetration of nanosystems

To evaluate the tumor penetration ability of the PPD/PDP nanosystem in A2780 multicellular spheroids (MCSs), A2780 cells were first cultured in RPMI-1640 medium supplemented with 10% FBS until the logarithmic growth phase. Cells were seeded at a density of 5000 cells per well in ultra-low attachment plates and incubated at 37 °C with 5% CO₂ for 3–5 days to form stable spheroids. The culture medium pH was adjusted to 7.4 or 6.8 before adding fluorescently labeled PPD/PDP@si-FDX1 nanomicelles (50 µg/mL). Incubation was performed for 4–12 h with gentle shaking to ensure uniform exposure. After incubation, spheroids were washed three times with PBS to remove uninternalized nanomicelles, fixed with 4% paraformaldehyde, and stained with DAPI to visualize nuclei. Confocal laser scanning microscopy (CLSM) was conducted with excitation wavelengths of 405 nm for DAPI and 350 nm for Hoechst 33,342-labeled si-FDX1. ImageJ software was used to quantify and analyze the depth of nanosystem penetration under different pH conditions.

### In *Vitro* Cellular Uptake of Nanomicelles

A2780 cells were seeded at 1 × 10⁵ cells per well in confocal dishes and 6-well plates and cultured in DMEM adjusted to pH 7.4 or 6.8. When cells reached 60–70% confluency, si-FDX1 (30 nM) and PPD/PDP@si-FDX1 (equivalent to 98.6 µg/mL PPD/PDP micelles containing the same siRNA amount) were added. Cells were co-cultured with micelle suspensions for 12 h. For qualitative analysis, cells in confocal dishes were fixed with 4% paraformaldehyde, permeabilized with 0.2% Triton X-100, and stained with a red nuclear dye. The intracellular distribution and uptake efficiency of the nanomicelles were visualized using a CLSM (LSM 510 META, Olympus, Japan). For quantitative evaluation, cells in 6-well plates were washed with PBS, harvested by centrifugation (2000 rpm, 10 min, 4 °C), resuspended in binding buffer, and analyzed using flow cytometry (FCM; BD Biosciences, USA).

### In *Vivo* Animal Experiments

Thirty female C57BL/6 mice (5–6 weeks old) were obtained from Hunan SJA Laboratory Animal Co., Ltd. The mice were maintained under controlled conditions at 22 ± 2 °C with 55 ± 5% relative humidity and a 12-hour light/dark cycle, with free access to food and water. All experimental procedures complied with the guidelines of the U.S. National Institutes of Health and were approved by the institutional Animal Ethics Committee.

To establish the tumor-bearing model, chemoresistant SKOV3-TR cells (1 × 10⁶) were subcutaneously injected into the right inguinal region of each mouse. When tumor volumes reached approximately 50 mm³, the mice were randomly assigned to four groups (*n* = 6 per group): saline, si-FDX1, PPD/TD, and PPD/PDP@si-FDX1. All groups received concurrent paclitaxel (TAX) treatment. Injections were administered intravenously three times per week for 18 days, with siRNA at 0.5 mg/kg and TAX at 2 mg/kg [27].

For biodistribution analysis, si-FDX1 was labeled with Cy5 (MM50114, Maokang Biotechnology, Shanghai, China). Mice in the si-FDX1 (*n* = 3) and PPD/PDP@si-FDX1 (*n* = 3) groups were euthanized 24 h after injection. Major organs (liver, kidneys, spleen, heart, lungs, and tumors) were excised for fluorescence imaging using an in vivo imaging system (LB983, Berthold Technologies, Germany). Blood samples were collected at several time points within 24 h post-injection, plasma was separated, and fluorescence intensity was quantified to generate time-dependent distribution curves.

Mouse body weight and tumor volume were recorded every two days. Tumor volume was calculated using the formula: tumor volume = ab^2^/2, where a is the longest diameter and b is the shortest diameter. After the final treatment, mice were monitored for an additional 15 days. All mice were euthanized 15 days post-treatment, and tumors and major organs were collected for histological and pathological analysis, including hematoxylin and eosin (H&E) staining, TUNEL assay, and immunofluorescence staining.

### H&E staining

H&E staining was performed using the H&E Staining Kit (C0105S, Beyotime, China) to assess histopathological alterations in the heart, liver, spleen, lungs, kidneys, and tumor tissues. Tissue samples were fixed in 4% paraformaldehyde, dehydrated, and cleared before paraffin embedding. Sections were cut to a thickness of 5 μm using a microtome, deparaffinized, and rehydrated. The slides were then stained with hematoxylin, washed with distilled water, and briefly dipped in 95% ethanol. Subsequently, sections were counterstained with eosin, differentiated in 70% ethanol containing hydrochloric acid, dehydrated, cleared, and mounted using neutral resin. Morphological changes were observed and recorded under a light microscope.

### TUNEL assay

Apoptotic cells in mouse tumor tissues were detected using the TUNEL Apoptosis Assay Kit (C1091, Beyotime, China). Paraffin-embedded sections were dewaxed in xylene and rehydrated through a graded ethanol series. After blocking with goat serum at ambient temperature, sections were subjected to TUNEL staining. Cell nuclei were counterstained with DAPI, and the sections were observed using a CLSM.

### Statistical analysis

All experiments were conducted independently at least three times. Data are presented as mean ± standard deviation (SD). Statistical comparisons between two groups were performed using independent-sample t-tests, while multiple-group comparisons were analyzed using one-way analysis of variance (ANOVA). When significant differences were detected, Tukey’s HSD post hoc test was applied for pairwise comparisons. For non-normally distributed data or unequal variances, the Mann–Whitney U test or Kruskal–Wallis H test was used. All statistical analyses were performed using GraphPad Prism 8.0, with *p* < 0.05 considered statistically significant.

## Results

### FDX1 enhances copper levels and promotes TAX resistance in OC cells

**Fig. 1:**
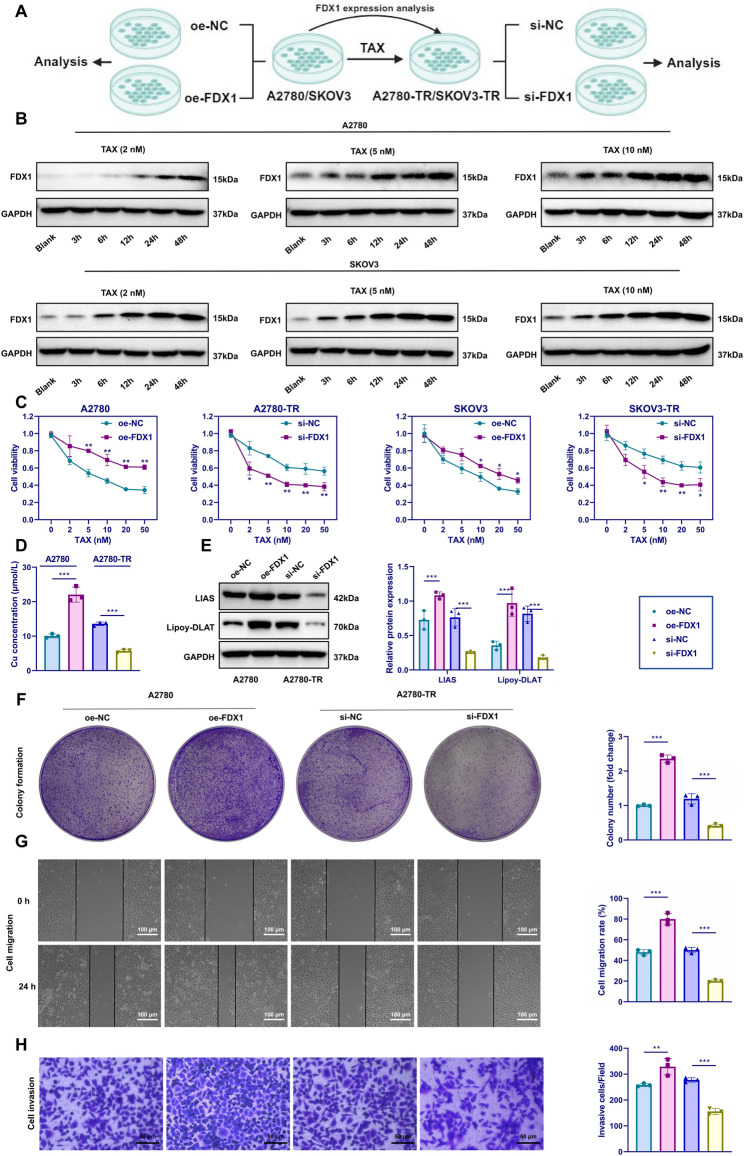
Effects of FDX1 on OC cell proliferation, migration, invasion, and TAX resistance. Note: **A** Schematic of experimental procedures; **B** Western Blot analysis of FDX1 protein expression in A2780 and SKOV3 cells treated with varying TAX concentrations over different time points; **C** Cell viability assessed by CCK-8 assay in different groups; **D** Copper ion content in different groups; **E** Western Blot analysis of LIAS and Lipoy-DLAT protein expression in each group; **F** Colony formation assay to evaluate cell proliferation in different groups; **G** Scratch assay to measure cell migration capability, Scale bar = 100 μm; **H** Transwell assay for evaluating cell invasion capability, Scale bar = 50 μm. *p < 0.05, **p < 0.01, ***p < 0.001. Experiments were repeated three times

Integrated RNA-seq and proteomic analyses based on TCGA, GTEx, and CPTAC datasets revealed significantly higher mRNA and protein expression levels of FDX1 in OC tissues compared with normal ovarian tissues (Figure S1A-B). To model drug resistance, the parental OC cell lines A2780 and SKOV3 were exposed to stepwise increasing concentrations of paclitaxel (TAX), establishing the resistant sublines A2780-TR and SKOV3-TR (Fig. 1A). As shown in Figure S1C, the dose–response curves of both resistant lines shifted notably to the right (Figure S1C), accompanied by higher IC₅₀ values and resistance indices, confirming the successful development of TAX-resistant sublines. Subsequent RT-qPCR and WB analyses demonstrated that FDX1 expression was markedly elevated in A2780-TR and SKOV3-TR cells compared with their parental counterparts (Figure S1D-E). To assess whether cuproptosis contributed to this phenotype, DLAT oligomerization was analyzed as a molecular marker, following established methods [28, 29]. The expression of LIAS and Lipoy-DLAT, along with intracellular copper ion concentrations, was measured by WB and assay kits. Resistant cells exhibited significantly higher copper levels and expression of cuproptosis-related proteins (Figure S1F-G), suggesting a crucial role of FDX1-induced cuproptosis in OC TAX resistance.

We exposed A2780 and SKOV3 cells to various TAX concentrations (2, 5, and 10 nM) and over different durations (3, 6, 12, 24, and 48 h). WB analysis of FDX1 post-treatment revealed a significant increase in protein expression after 12, 24, and 48 h in both cell lines (Fig. 1B). Consistent with previous findings [30], upregulation of FDX1 may represent an adaptive response that preserves mitochondrial stability under copper stress, explaining its higher expression in OC and resistant cells.

Functional experiments were conducted by silencing FDX1 in A2780-TR and SKOV3-TR cells and overexpressing FDX1 in A2780 and SKOV3 cells. Among three siRNA sequences targeting FDX1, the most effective one was selected (Figure S1H), and overexpression efficiency was confirmed (Figure S1I). CCK-8 assays revealed that FDX1 overexpression enhanced cell viability following TAX exposure, whereas silencing FDX1 had the opposite effect (Fig. 1C). These findings indicate that FDX1 promotes TAX resistance in OC cells.

Further, 10 nM TAX treatment resulted in elevated copper ion levels and increased expression of LIAS and Lipoy-DLAT in FDX1-overexpressing cells, while FDX1 knockdown produced the opposite effect (Fig. 1D-E; Figure S2A-B). In clonogenic assays, FDX1 overexpression significantly enhanced colony formation, while silencing suppressed it. Similarly, scratch and Transwell assays demonstrated increased migration and invasion in the FDX1-overexpressing group and reduced motility in the knockdown group (Fig. 1F-H; Figure S2C-E). Collectively, these results demonstrate that FDX1 promotes copper accumulation, enhances cuproptosis-related protein expression, and strengthens the proliferative, migratory, and invasive capacities of OC cells.

### Cuproptosis-Related gene FDX1 enhances TAX resistance in OC cells by promoting autophagy

**Fig. 2:**
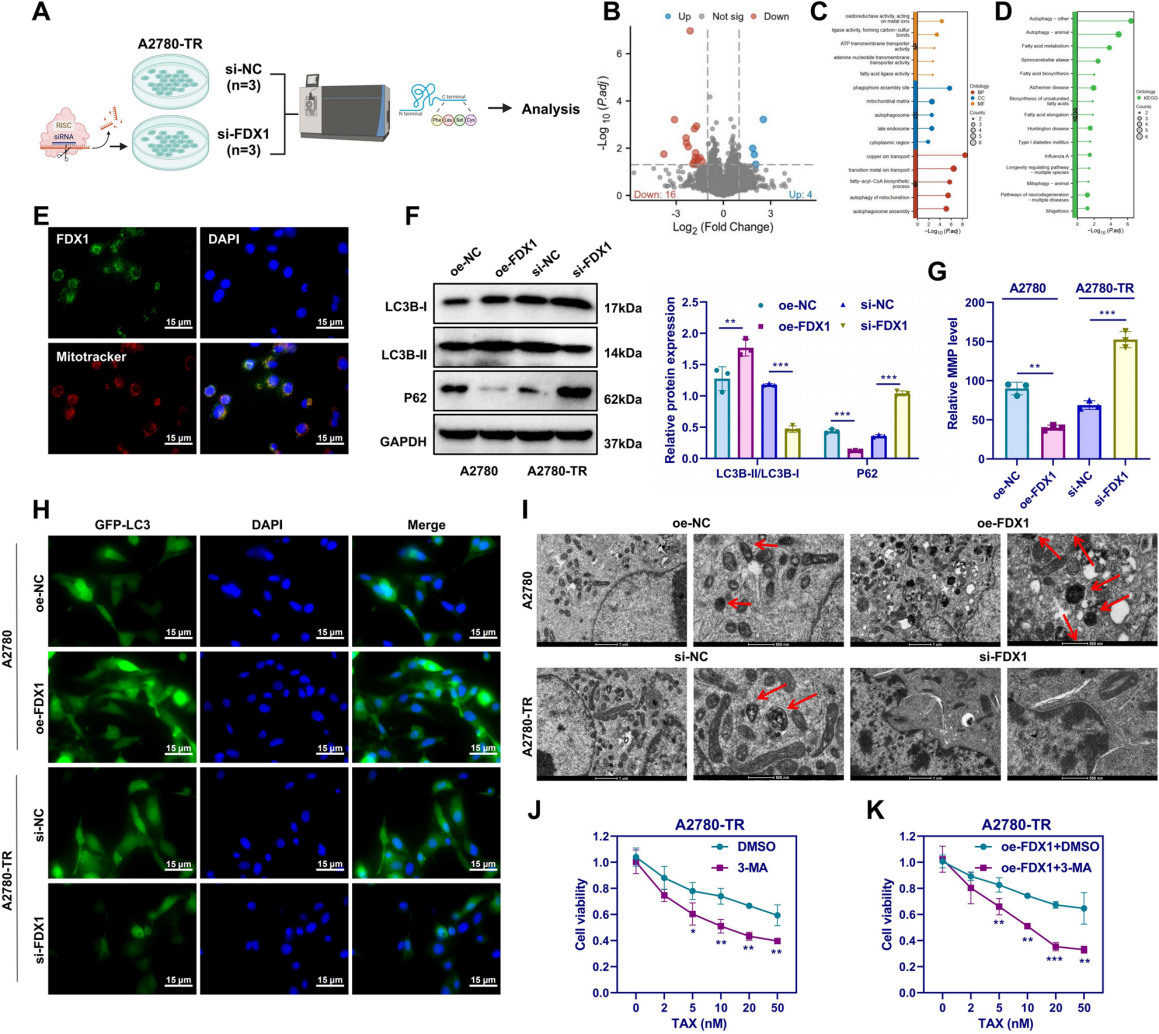
Effects of FDX1 on autophagy and TAX resistance in A2780 cells. Note: **A** Schematic of proteomic analysis workflow; **B** Volcano plot of differential expression analysis between si-NC (*n* = 3) and si-FDX1 (*n* = 3) groups in A2780-TR cells; **C** Lollipop plot of GO enrichment analysis results for DEPs. **D** Lollipop plot of KEGG enrichment analysis of DEPs; **E** Mitochondria labeled with MitoTracker (red), with FDX1 labeled in green; white arrows indicate representative mitochondria stained by FDX1 and MitoTracker in A2780-TR cells, Scale bars = 15 μm; **F** Western blot analysis of LC3B-I, LC3B-II, and P62 protein expression in each group of cells following treatment with 10 nM TAX for 48 h; **G** MMP levels detected by JC-1 assay; **H** Immunofluorescence staining of LC3-positive cells, Scale bar = 15 μm; **I** TEM images showing mitochondrial morphology in each group, Scale bar = 1 μm (left), 500 nm (right); red arrows indicate autophagosomes; **J** CCK-8 assay for assessing cell viability in DMSO and 3-MA treatment groups; **K** CCK-8 assay to evaluate cell viability in oe-FDX1 + DMSO and oe-FDX1 + 3-MA groups. *p < 0.05, **p < 0.01, ***p < 0.001. Experiments were repeated three times

To investigate the mechanism by which FDX1 influences TAX resistance in OC cells, proteomic analysis was performed on A2780-TR cells transfected with si-NC (*n* = 3) or si-FDX1 (*n* = 3) (Fig. 2A). DEPs were defined as those with |log₂FC| >1 and *p* < 0.05, identifying 16 significantly downregulated and 4 upregulated DEPs (Fig. 2B). Gene ontology (GO) enrichment revealed that DEPs were primarily involved in biological processes (BP) such as copper ion transport, metal ion homeostasis, fatty acyl-CoA biosynthesis, mitophagy, and autophagosome assembly. Enriched cellular components (CC) included phagophore assembly sites, mitochondrial matrix, cytoplasm, autophagosomes, and late endosomes. Molecular functions (MF) associated with DEPs included ATP transmembrane transport, metal ion-binding oxidoreductase activity, and fatty acid ligase activity. KEGG pathway analysis indicated significant enrichment in autophagy and fatty acid metabolism pathways (Fig. 2C-D). Immunofluorescence imaging showed that FDX1 predominantly localized to the cytoplasm and co-localized with mitochondria labeled by MitoTracker Red (Fig. 2E). Prior studies have reported a strong association between FDX1 and autophagy, particularly under stress conditions such as drug exposure [31]. These findings suggested that FDX1 might regulate autophagy to modulate TAX resistance in OC cells.

To determine whether autophagy was activated during TAX treatment, A2780-TR and SKOV3-TR cells were treated with 10 nM TAX in the presence or absence of 10 nM BafA1 for 24 h. WB was performed to analyze LC3B and P62 expression, immunofluorescence was used to visualize LC3B localization, and TEM was employed to observe autophagosome formation. TAX treatment led to an increase in LC3B expression and a decrease in P62 levels, with both markers accumulating upon BafA1 co-treatment (Figure S3A). The number of GFP-LC3 puncta also increased significantly after TAX exposure and further accumulated in the presence of BafA1 (Figure S3B). TEM confirmed these findings (Figure S3C). These results suggest that TAX induces autophagosome formation and increases autophagic flux in OC cells.

Next, to assess the role of FDX1 in autophagy regulation, A2780-TR and SKOV3-TR cells with silenced or overexpressed FDX1 were exposed to 10 nM TAX for 48 h. In both cell lines, FDX1 overexpression significantly increased LC3B-II levels, accelerated P62 degradation (Fig. 2F; Figure S4A), reduced MMP (Fig. 2G; Figure S4B), enhanced LC3-positive puncta formation (Fig. 2H; Figure S4C), and promoted autophagosome generation (Fig. 2I; Figure S4D). In contrast, silencing FDX1 produced opposite effects. These findings indicate that FDX1 significantly enhances autophagic activity in TAX-treated OC cells.

To further confirm the link between FDX1 and autophagy-mediated TAX resistance, the autophagy inhibitor 3-methyladenine (3-MA) was applied to OC cells. Inhibition of autophagy with 3-MA significantly reduced cell viability compared with the DMSO control (Fig. 2J; Figure S4E). Furthermore, relative to the oe-FDX1 + DMSO group, the oe-FDX1 + 3-MA group exhibited a marked decrease in cell viability (Fig. 2K; Figure S4F). These findings demonstrate that FDX1 overexpression enhances TAX resistance in OC cells by promoting autophagy.

### FDX1 promotes autophagosome formation by activating the ULK1/ATG13 axis

Previous research has shown that copper is essential for the activity of the autophagy-initiating kinase ULK1, as copper directly binds to ULK1 to promote ATG13 phosphorylation and autophagosome formation, ultimately enhancing tumor growth [16]. We hypothesized that silencing FDX1 might reduce copper levels, thereby inhibiting the activation of the ULK1-ATG13 axis and decreasing autophagosome formation.

**Fig. 3:**
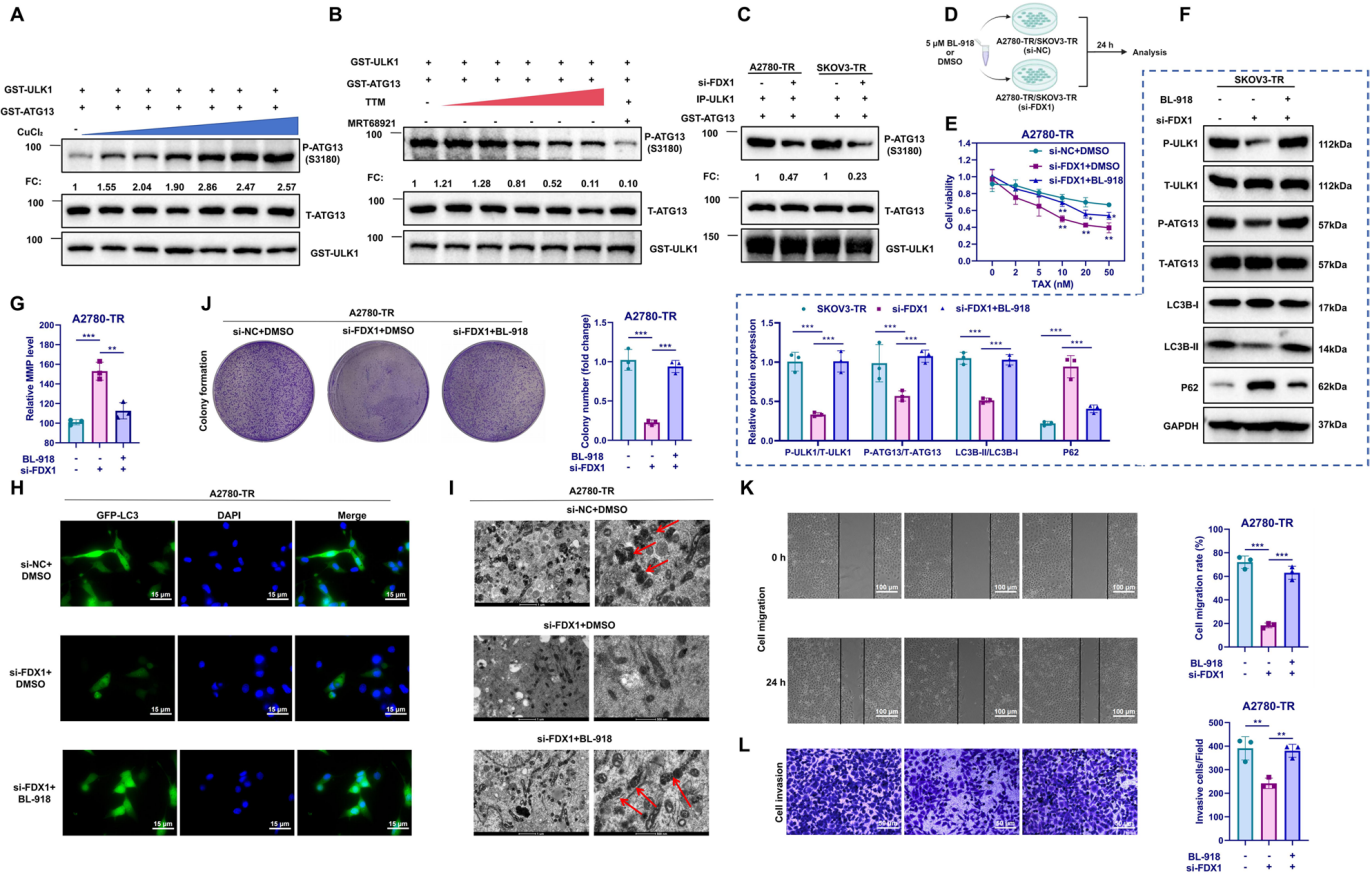
Mechanistic investigation of FDX1’s influence on autophagy and TAX resistance in A2780 cells. Note: **A** Western Blot analysis of P-ATG13, T-ATG13, and GST-ULK1 expression as CuCl_2_ concentration increases from 0 µM to 10 µM; **B** Western Blot analysis of P-ATG13, T-ATG13, and GST-ULK1 expression with TTM concentration ranging from 0 to 50 µM or after treatment with 10 µM MRT68921; **C** IP analysis of P-ATG13, T-ATG13, and IP-ULK1 (ULK1 protein pulled down during IP); **D** Schematic diagram of the experimental procedures; **E** CCK-8 assay to assess cell viability across groups; **F** Western Blot analysis of ULK1, ATG13, LC3B-I, LC3B-II, and P62 protein expression in each group; **G** JC-1 assay to evaluate MMP levels in different groups; **H** Immunofluorescence staining to detect LC3-positive cells in each group; **I** TEM images showing mitochondrial morphology in A2780 cells, Scale bar = 1 μm (left), 500 nm (right), with red arrows indicating autophagosomes; **J** Colony formation assay to evaluate the proliferative capacity of each group; **K** Scratch assay to assess cell migration capability, Scale bar = 100 μm; **L** Transwell assay to evaluate cell invasion capability, Scale bar = 50 μm. *p < 0.05, **p < 0.01, ***p < 0.001. Experiments were repeated three times

To validate this, recombinant protein assays demonstrated that increasing concentrations of CuCl₂ led to a marked rise in ATG13 phosphorylation, while total ATG13 protein levels remained unchanged (Fig. 3A). Conversely, treatment with the copper chelator TTM resulted in a dose-dependent reduction of ATG13 phosphorylation (Fig. 3B). Inhibition of ULK1/2 with MRT68921 nearly abolished ATG13 phosphorylation, confirming that ULK1 kinase activity is essential for this process (Fig. 3B). Immunoprecipitation assays of ULK1 complexes from FDX1-silenced A2780-TR and SKOV3-TR cells showed a substantial decrease in ATG13 phosphorylation compared with controls, indicating that FDX1 maintains ULK1 activity via copper regulation (Fig. 3C).

To further clarify the role of FDX1 in regulating autophagy, the ULK1 activator BL-918 (10 µM, 24 h) was applied to FDX1-silenced A2780-TR and SKOV3-TR cells. Treatment with BL-918 alone (without BafA1) increased LC3-II levels, reduced LC3-I, and decreased p62 expression, indicating autophagy activation (Figure S5A–B). When BafA1 (50–80 nM, 2–4 h) was subsequently added, LC3-II further accumulated while p62 levels rose, consistent with lysosomal inhibition. These results suggest that BL-918 restores autophagic activity suppressed by FDX1 knockdown. To verify that FDX1 regulates ULK1 and ATG13 phosphorylation through copper modulation, and to evaluate the connection between the ULK1/ATG13 axis and TAX resistance, the phosphorylation levels of ULK1 and ATG13 were analyzed in TAX-resistant and FDX1-overexpressing cells treated with the copper chelator TTM (Figure S5C). Compared with SKOV3 cells, SKOV3-TR cells exhibited markedly elevated phosphorylation of ULK1 and ATG13 (Figure D). Moreover, in FDX1-overexpressing cells, TTM treatment significantly decreased ATG13 phosphorylation relative to DMSO controls (Figure S5E). These results indicate that FDX1 overexpression promotes ATG13 phosphorylation by increasing copper levels.

To further explore the mechanism, FDX1-silenced cells were treated with BL-918, and autophagy- and resistance-related markers were examined (Fig. 3D). Compared with the si-NC + DMSO group, the si-FDX1 + DMSO group displayed markedly reduced phosphorylation of ULK1 and ATG13, decreased LC3B-II levels, slower P62 degradation, elevated MMP, and diminished autophagosome formation. In contrast, the si-FDX1 + BL-918 group exhibited significantly increased ATG13 phosphorylation and restored autophagic activity (Fig. 3F-I; Figure S6B-E). These results indicate that activation of ULK1 effectively reversed the suppression of autophagosome formation and autophagic flux caused by FDX1 knockdown. Functionally, BL-918 treatment also reversed the decrease in TAX resistance induced by FDX1 silencing. Relative to the si-FDX1 + DMSO group, cells in the si-FDX1 + BL-918 group showed decreased viability but increased proliferative, migratory, and invasive capacities (Fig. 3E and J-L; Figure S6A; Figure S6F-H).

These findings demonstrate that FDX1 overexpression increases copper ion levels, activates ULK1, and promotes ATG13 phosphorylation and autophagic activity. Inhibition of ULK1 or copper chelation effectively counteracts these effects.

### Successful Preparation of PPD/PDP@si-FDX1 nanomicelles

The TME, characterized by low pH and elevated ROS, often reduces drug efficacy and promotes the emergence of drug resistance. Given the established role of FDX1 in promoting TAX resistance in OC cells, a targeted nanodelivery system capable of silencing FDX1 was developed to enhance therapeutic outcomes. The objective of this study was to construct pH- and ROS-responsive nanomicelles for the co-delivery of si-FDX1 to overcome TAX resistance in OC.

**Fig. 4:**
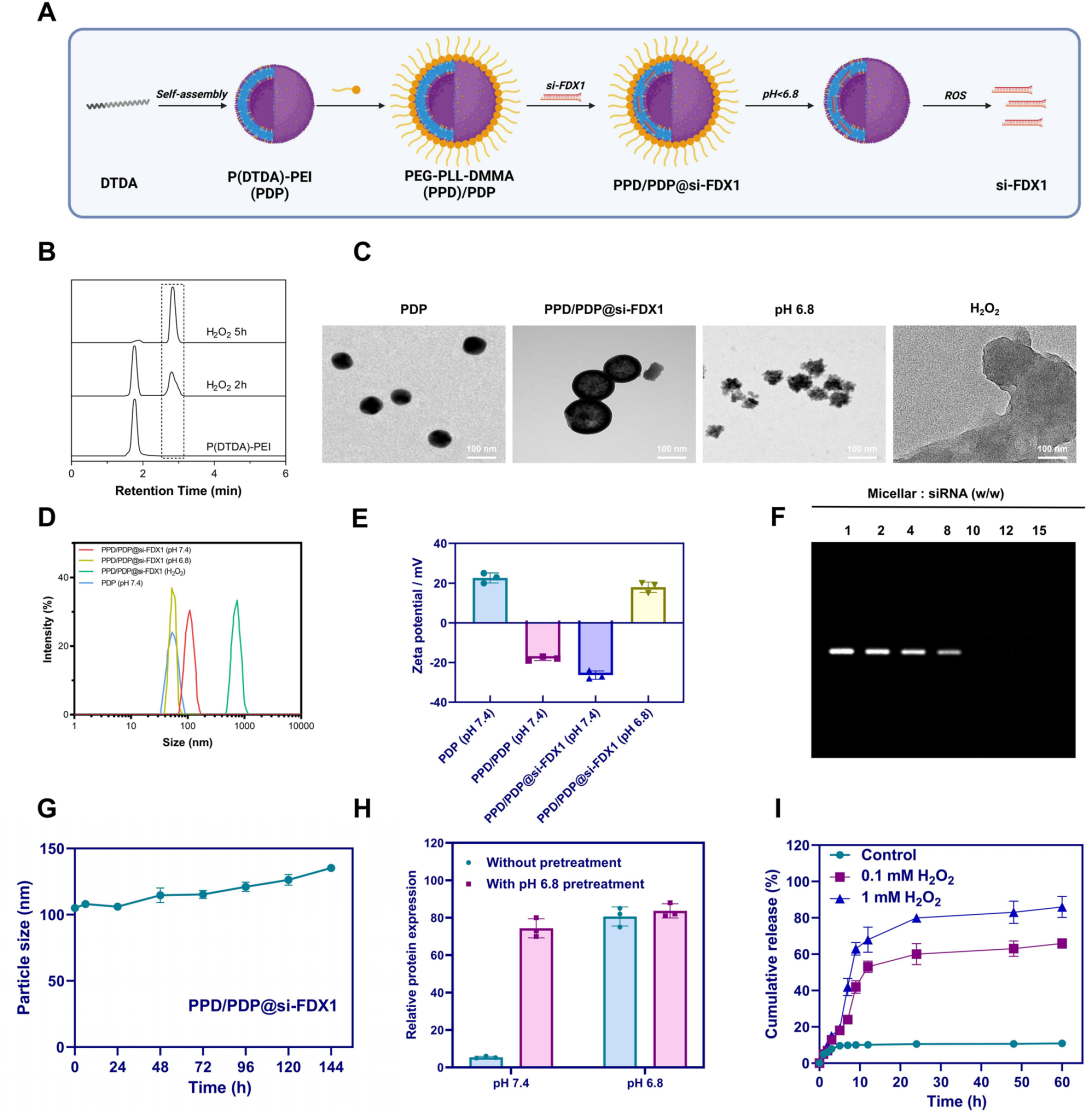
Preparation, characterization, and pH/ROS responsiveness of PPD/PDP@si-FDX1. Note: **A** Schematic illustration of nanomicelle synthesis; **B** HPLC chromatograms of free TAX and PDP prodrug polymers incubated in a MeOH/H_2_O (4:1, v/v) solution containing 0.1 mM H_2_O_2_; **C** TEM images of PDP and PPD/PDP@si-FDX1, as well as TEM images of PPD/PDP@si-FDX1 after incubation for 2 h under pH 6.8 and H_2_O_2_ treatment, Scale bar = 100 nm; **D** DLS data of PDP and PPD/PDP@si-FDX1 in various groups; **E** Zeta potential measurements of PDP and PPD/PDP@si-FDX1 in each group; **F** Gel electrophoresis analysis of PPD/PDP@si-FDX1 binding affinity; **G** Stability of PPD/PDP@si-FDX1 after 6 days of incubation in 10% serum; **H** BSA adsorption capacity of micelles with or without pre-treatment at pH 6.8; **I** si-FDX1 release efficiency of PPD/PDP@si-FDX1 after treatment with varying concentrations of H_2_O_2_

The shell copolymer (PPD) was synthesized by conjugating DMMA to PEG-PLL, which was obtained through ring-opening polymerization of Lys-NCA followed by removal of benzoxycarbonyl groups from PEG-PLLZ. The core copolymer (PDP) was prepared by incorporating branched PEI into the P(DTDA) copolymer. Through self-assembly and electrostatic interactions, the PPD/PDP@si-FDX1 micelle complex was successfully formed, consisting of a PDP core and a PPD shell (Fig. 4A).

The ROS sensitivity of the PDP copolymer was confirmed using HPLC analysis. Following H₂O₂ exposure, the initial PDP peak intensity decreased markedly, indicating oxidative cleavage and efficient disassembly under ROS-rich conditions (Fig. 4B). TEM and dynamic light scattering (DLS) characterization revealed that PDP and PPD/PDP@si-FDX1 micelles exhibited uniform, spherical morphology with a distinct core–shell structure. Incorporation of siRNA increased the micelle diameter from 48 nm to approximately 100 nm (Fig. 4C-D), confirming successful siRNA loading and shell formation. Furthermore, zeta potential measurements showed a reduction in surface charge from 24 mV to −16 mV and − 31 mV (Fig. 4E), further confirming the successful construction of the PPD/PDP@si-FDX1 nanosystem.

The biological stability and siRNA encapsulation efficiency of the PPD/PDP@si-FDX1 nanomicelles were assessed using a gel retardation assay. As the mass ratio of PPD/PDP to siRNA increased, siRNA loading efficiency improved markedly. The resulting micelles exhibited an average diameter of approximately 100 nm and a surface potential of − 31 mV (Fig. 4E-F), indicating efficient siRNA encapsulation. Furthermore, after incubation in 10% serum, the size of PPD/PDP@si-FDX1 micelles remained stable, demonstrating good biological stability (Fig. 4G), which is critical for systemic siRNA delivery.

In pH-responsive experiments, we found that when the pH decreased from 7.4 to 6.8, the micelle size reduced from approximately 100 nm to 52 nm, indicating the successful removal of the PPD shell (Fig. 4C-D). Zeta potential measurements showed that at pH 6.8, the micelle charge reversed from − 31 mV to 20 mV (Fig. 4E), indicating surface charge conversion. Additionally, under pH 6.8 conditions, the adsorption of BSA on the micelle surface significantly increased to approximately 78%, compared to only 8% at pH 7.4 (Fig. 4H), suggesting that charge reversal under acidic conditions enhances cellular uptake efficiency.

In ROS-responsive experiments, the ROS sensitivity of the nanomicelles was further verified through TEM, DLS, UV–Vis spectroscopy, and real-time drug release assays. After exposure to 0.1 mM H₂O₂, TEM images showed a transition from intact spherical micelles to disrupted structures, and DLS analysis revealed similar disassembly patterns (Fig. 4C-D). Quantitative release studies demonstrated that in the absence of H₂O₂, si-FDX1 release remained below 9% over 60 h, indicating high stability. However, with 0.1 mM and 1 mM H₂O₂, si-FDX1 release increased dramatically to 72% and 92%, respectively (Fig. 4I), demonstrating that the PDP copolymer effectively disassembles and releases si-FDX1 under ROS conditions.

In conclusion, PPD/PDP@si-FDX1 nanomicelles exhibit excellent pH/ROS responsiveness, facilitating precise drug delivery and controlled release within the TME.

### PPD/PDP@si-FDX1 nanomicelles improve tumor penetration and endocytosis of si-FDX1

**Fig. 5:**
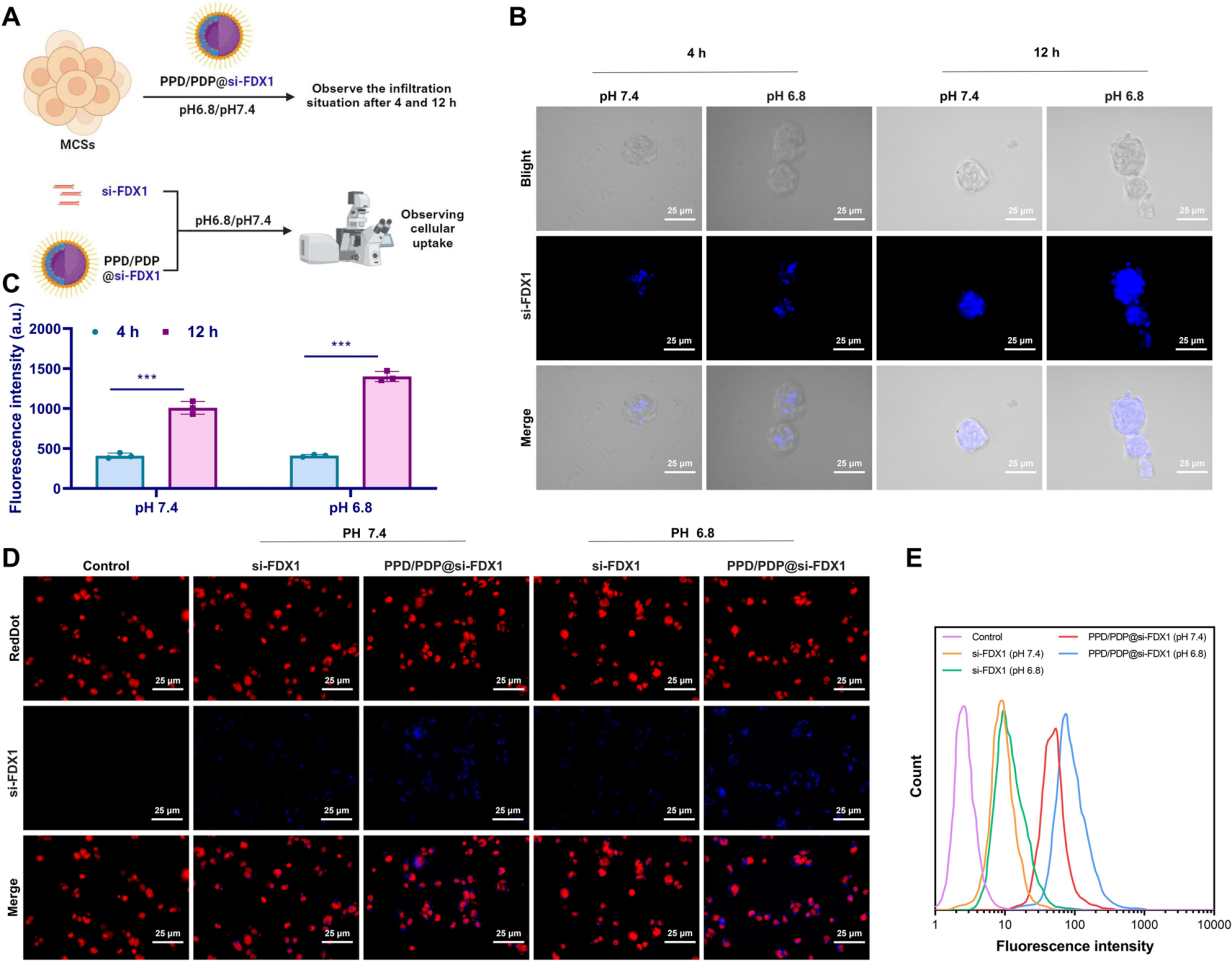
Tumor penetration and in vitro endocytosis of PPD/PDP@si-FDX1 Note: **A** Schematic of cell experimental procedures; **B**-**C** Tumor penetration of PPD/PDP@si-FDX1 in A2780 MCS after incubation for 4 h and 12 h at pH 7.4 and pH 6.8, Scale bar = 25 μm **B**, with quantification of fluorescence intensity **C**; **D**-**E** CLSM images of A2780 cells after 24 h of incubation with si-FDX1 and PPD/PDP@si-FDX1 under pH 7.4 and pH 6.8 conditions, Scale bar = 25 μm **D**, and quantitative FCM analysis results **E**. **p < 0.01, ***p < 0.001. Experiments were repeated three times

The tumor penetration and cellular internalization of PPD/PDP@si-FDX1 nanomicelles were systematically evaluated (Fig. 5A). Using A2780 MCSs, we investigated the tumor penetration capability of the PPD/PDP nanomicelles. At pH 7.4, blue fluorescence representing si-FDX1 was primarily confined to the outer layer of MCSs, indicating limited diffusion. In contrast, at pH 6.8, fluorescence intensity was markedly higher and distributed more uniformly throughout the spheroids, suggesting deeper micelle penetration. The degree of penetration increased with prolonged incubation up to 12 h (Fig. 5B-C).

Quantitative analysis by CLSM and FCM revealed that intracellular fluorescence intensity in cells treated with PPD/PDP@si-FDX1 was significantly greater than in those treated with free si-FDX1, under both neutral and acidic conditions (Fig. 5D). Moreover, the micelle-mediated uptake at pH 6.8 was substantially higher than at pH 7.4, as demonstrated by stronger fluorescence signals and confirmed by FCM quantification (Fig. 5E). These findings demonstrate that the PPD/PDP@si-FDX1 nanomicelles exhibit significantly enhanced tumor penetration and cellular uptake under acidic conditions (pH 6.8), establishing a foundation for targeting the TME effectively.

### PPD/PDP@si-FDX1 nanomicelles significantly inhibit autophagy in OC cells and further reduce TAX resistance

**Fig. 6:**
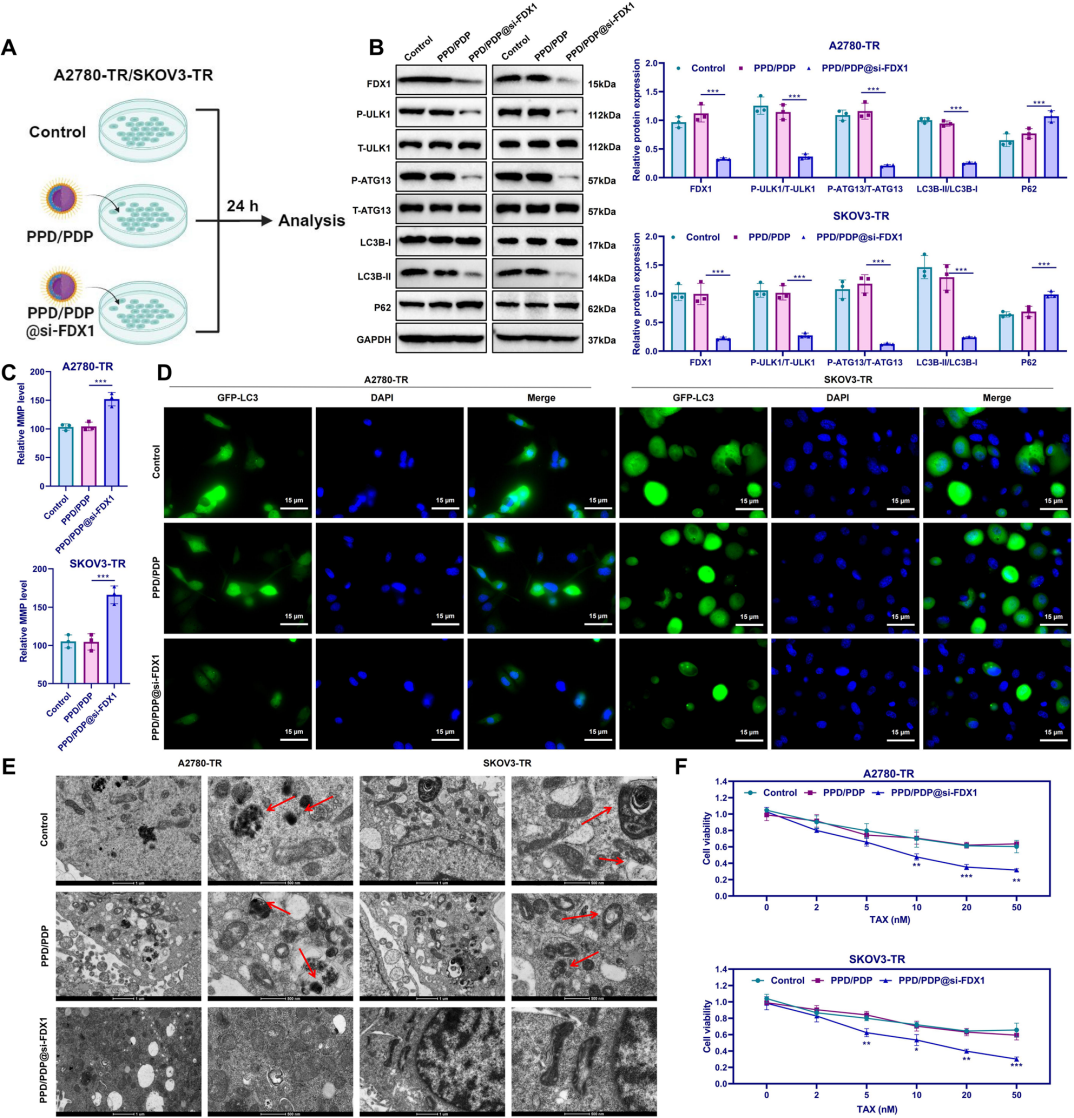
Effects of PPD/PDP@si-FDX1 on autophagy and TAX resistance in OC cells. Note: **A** Schematic of the cell experimental procedures; **B** Western Blot analysis of FDX1, ULK1, ATG13, LC3B-I, LC3B-II, and P62 protein expression in each group; **C** JC-1 assay to measure MMP levels across groups; **D** Immunofluorescence staining for LC3-positive expression in each group, Scale bar = 15 μm; **E** TEM images showing mitochondrial morphology in different groups, Scale bar = 1 μm (left) and 500 nm (right), with red arrows indicating autophagosomes; **F** CCK-8 assay to assess cell viability across groups. *p < 0.05, **p < 0.01, ***p < 0.001. Experiments were repeated three times

We treated A2780-TR and SKOV3-TR cells with PPD/PDP and PPD/PDP@si-FDX1 to investigate the effects of this nanosystem on autophagy and TAX resistance in OC cells (Fig. 6A). No significant differences were observed between the Control and PPD/PDP groups, indicating that the nanocarrier itself did not affect cellular function. However, compared with PPD/PDP, PPD/PDP@si-FDX1 treatment led to a marked downregulation of FDX1 protein expression, accompanied by decreased phosphorylation of ULK1 and ATG13, reduced LC3B-II levels, slower P62 degradation, increased MMP, and fewer autophagosomes (Fig. 6B-E). These findings confirm that PPD/PDP@si-FDX1 efficiently silences FDX1 and effectively suppresses autophagic activity in TAX-resistant OC cells. In addition, functional assays demonstrated that PPD/PDP@si-FDX1 significantly enhanced TAX sensitivity. Compared with the PPD/PDP group, PPD/PDP@si-FDX1-treated cells exhibited lower viability, reduced proliferation, and weakened migratory and invasive capabilities, while no notable difference was detected between the Control and PPD/PDP groups (Fig. 6F; Figure S7A-C).

Collectively, these results demonstrate that PPD/PDP@si-FDX1 nanomicelles suppress autophagy by silencing FDX1, thereby restoring TAX sensitivity in OC cells. The enhanced tumor penetration and targeted gene-silencing efficiency of the PPD/PDP nanosystem highlight its potential as an effective therapeutic platform to overcome TAX resistance and improve treatment outcomes in OC.

### PPD/PDP@si-FDX1 Exhibit Enhanced Antitumor Efficacy In Vivo

**Fig. 7:**
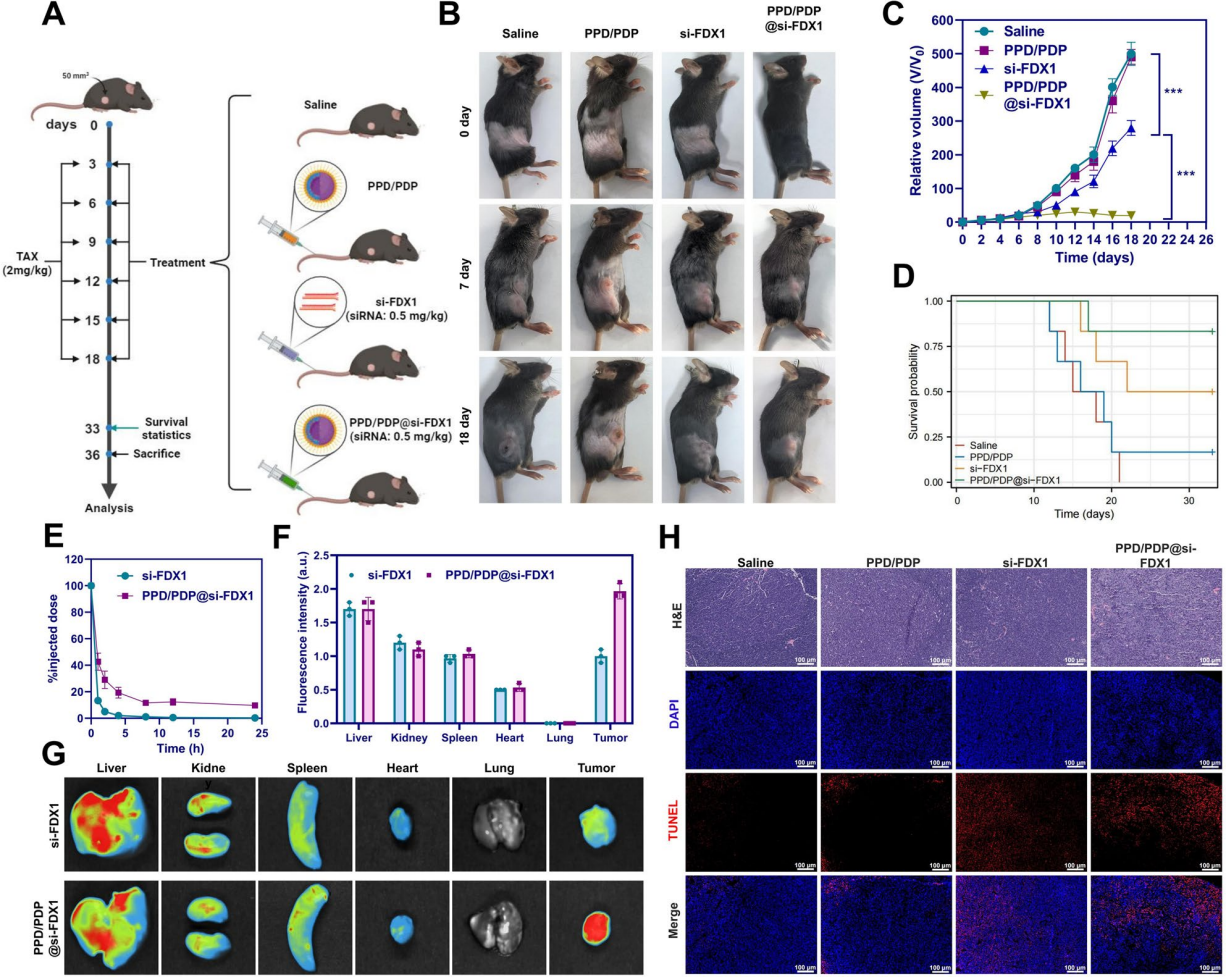
In *vivo* antitumor activity of PPD/PDP@si-FDX1 Note: **A** Schematic of the animal experimental procedures; **B** Tumor images of mice in each group at 0, 7, and 18 days post-treatment; **C** Tumor volumes in each group of mice; **D** Survival rates of mice across groups; **E** Pharmacokinetics of si-FDX1 after intravenous injection at 0.5 mg/kg in tumor-bearing mice at 24 h (*N* = 3); **F**-**G** Biodistribution of si-FDX1 in mice 24 h after intravenous injection (*N* = 3); **H** H&E and TUNEL staining images of tumors in each group, Scale bar = 100 μm. *p < 0.05, **p < 0.01, ***p < 0.001. Each animal group consisted of six mice

We established a subcutaneous tumor xenograft model and administered PPD/PDP, si-FDX1, and PPD/PDP@si-FDX1 via tail vein injection, using saline as the blank control (Saline group) and concurrently administering TAX treatment (Fig. 7A). Tumor growth in the Saline and PPD/PDP groups was comparable, confirming that the nanocarrier alone exerted no inhibitory effect. In contrast, si-FDX1 induced moderate tumor suppression, while PPD/PDP@si-FDX1 produced the strongest antitumor response (Fig. 7B-C). Furthermore, PPD/PDP@si-FDX1 significantly prolonged the survival of tumor-bearing mice compared to all other groups (Fig. 7D). Histological evaluation revealed no signs of tissue toxicity or organ damage (Figure S8), confirming the biocompatibility and safety of the nanosystem. Pharmacokinetic analysis showed that PPD/PDP@si-FDX1 exhibited a markedly longer blood circulation time compared to free si-FDX1 (Fig. 7E), attributable to the protective PEG-based PPD shell. Furthermore, the PPD/PDP@si-FDX1 nanosystem displayed significantly higher accumulation within tumor tissues than in other organs (Fig. 7F-G).

The in vivo antitumor performance of PPD/PDP@si-FDX1 nanomicelles was further evaluated using H&E and TUNEL staining of tumor tissues from the different treatment groups. No notable histopathological differences were detected between the Saline and PPD/PDP groups, confirming the biosafety of the carrier. In contrast, both si-FDX1 and PPD/PDP@si-FDX1 treatments induced varying degrees of tumor necrosis and apoptosis, with the strongest effects observed in the PPD/PDP@si-FDX1 group (Fig. 7H). These findings suggest that the PPD/PDP micelle significantly enhances drug delivery efficiency and bioavailability. Silencing FDX1 further boosts the antitumor effects of TAX and reduces its resistance. Overall, the PPD/PDP@si-FDX1 nanosystem demonstrates superior antitumor efficacy in vivo.

The in vivo silencing efficacy of PPD/PDP@si-FDX1 was further validated through immunofluorescence and WB analyses, alongside measurements of autophagy-related protein expression and copper ion levels in tumor tissues. Both the si-FDX1 and PPD/PDP@si-FDX1 treatment groups exhibited marked downregulation of FDX1, P-ULK1, P-ATG13, and LC3B-II/I, accompanied by a significant upregulation of P62 compared to the Saline group (Figure S9A-C). A significant reduction in cuproptosis levels was observed in both the si-FDX1 and PPD/PDP@si-FDX1 groups (Figure S9D). These findings were consistent with the in vitro results and confirmed that the antitumor activity of PPD/PDP@si-FDX1 is closely linked to copper regulation and autophagy inhibition mediated through the ULK1–ATG13 signaling axis. Compared with the si-FDX1 group, the PPD/PDP@si-FDX1 treatment led to a more pronounced downregulation of FDX1, P-ULK1, P-ATG13, and LC3B-II/I, alongside a marked increase in P62 expression (Figure S9A-C). Additionally, cuproptosis levels declined further in this group (Figure S9D). Collectively, these results demonstrate that the PPD/PDP nanosystem enhances the bioavailability and silencing efficiency of si-FDX1, resulting in greater inhibition of autophagy and copper-induced signaling.

## Discussion

In OC treatment, resistance to TAX presents a significant challenge, significantly limiting the long-term effectiveness of chemotherapy [32]. FDX1 significantly influences TAX resistance through its role in copper metabolism, affecting intracellular autophagy [14, 15, 17]. Previous studies have shown that FDX1 modulates multiple biological processes by controlling intracellular copper ion levels, including pathways involved in energy metabolism, oxidative stress, and protein lipoylation. Among these, autophagy has been identified as a key downstream process directly linked to chemoresistance [16, 33]. In the present study, FDX1 was found to regulate the autophagy pathway primarily through activation of the ULK1/ATG13 signaling axis, a mechanism that has been rarely reported in previous research. By exploring the complex relationship between FDX1 and TAX resistance, this study provides new theoretical insights and experimental support for the potential application of FDX1 in OC treatment.

Autophagy is a fundamental intracellular degradation process that plays a central role in cellular adaptation to chemotherapy stress and the development of drug resistance [11]. Exposure to chemotherapeutic agents induces oxidative stress and cellular damage, which activate the autophagy pathway. Through the degradation and recycling of key biomolecules and energy sources, tumor cells maintain metabolic homeostasis and recover proliferative capacity under cytotoxic stress [34, 35]. This activation enables cancer cells to evade chemotherapy-induced apoptosis and sustain survival, ultimately leading to therapeutic failure [36, 37]. Therefore, understanding the mechanisms of autophagy under chemotherapy stress and its contributions to resistance formation is crucial for developing new anticancer strategies and overcoming resistance in existing treatments. Compared with previous studies [38], chemotherapy-induced stress has been shown to upregulate autophagy in multiple types of tumors, facilitating cell survival under cytotoxic pressure through substrate degradation and recycling. This phenomenon has been widely discussed in mechanistic reviews and OC–specific studies. Consequently, therapeutic approaches combining chemotherapy with autophagy inhibition have been widely explored and reported to enhance anticancer efficacy [37]. However, in the context of OC, studies that incorporate copper homeostasis or copper signaling into this mechanistic framework, and further position FDX1 upstream of the autophagy initiation complex (ULK1/ATG13) for causal validation, remain limited.

FDX1 serves as a central mediator linking copper metabolism and autophagy. As a key regulator of cuproptosis, FDX1 governs intracellular copper reduction and trafficking, coupling copper ions to lipoylated TCA-cycle proteins. In parallel, copper functions as a noncanonical kinase cofactor that can directly modulate ULK1/2 activity, thereby integrating copper homeostasis with autophagy initiation. In the present study, both gain- and loss-of-function experiments demonstrated that altering FDX1 expression markedly affected autophagic activity, extending its functional scope beyond its previously recognized role in Fe–S cluster biogenesis. The findings indicate that FDX1 promotes autophagosome formation and directly regulates the autophagy pathway through activation of the ULK1/ATG13 axis. This mechanistic insight expands the current understanding of FDX1’s biological functions and emphasizes its significance in TAX resistance. Targeting FDX1 represents a promising approach for overcoming chemoresistance and improving therapeutic outcomes in OC.

Nanotechnology has advanced substantially in cancer therapy, particularly in the development of precise drug delivery systems [39–41]. In this study, pH/ROS-responsive nanomicelles were designed to exploit the acidic and oxidative characteristics of the TME, enabling controlled and site-specific drug release. The nanomicelles effectively delivered si-FDX1 to tumor cells, achieving high local drug concentrations and enhancing therapeutic efficacy through environment-responsive release. Compared with conventional delivery systems, these nanomicelles demonstrated greater targeting precision and superior release control. The successful construction and validation of this platform provide a foundation for applying similar nanostructures to other therapy-resistant cancers, underscoring the broad potential of nanotechnology in precision oncology.

A comprehensive series of in vitro and in vivo experiments confirmed the efficacy of FDX1-targeted nanomicelles in reversing TAX resistance. Compared with previous studies, the findings demonstrated a pronounced capacity to enhance the therapeutic effects of chemotherapy by precisely modulating copper metabolism and autophagy pathways. Targeting FDX1 markedly improved the antitumor activity of TAX and enhanced treatment outcomes by promoting drug penetration into tumor tissues and facilitating cellular uptake. These results provide a robust scientific foundation for further refinement of the nanodelivery platform and establish its potential for clinical translation. The data collectively suggest that FDX1-targeted nanomicelles represent a promising therapeutic approach, particularly for cancers exhibiting resistance to conventional chemotherapy.

The study employed A2780 and SKOV3 cell lines, along with a subcutaneous xenograft mouse model, to evaluate the therapeutic efficacy of FDX1-targeted nanomicelles. The A2780 and SKOV3 models effectively reproduce the biological characteristics and TAX-resistance profiles of OC in vitro, while the xenograft model provides a reliable in vivo system to simulate tumor growth and assess drug response. Using these complementary models ensured experimental rigor, reproducibility, and translational relevance. Moreover, the choice of models is consistent with prior OC research, facilitating direct comparison with existing literature and underscoring the superior capacity of FDX1-targeted nanomicelles to reverse TAX resistance.

The study identified the central role of the FDX1–ULK1/ATG13–autophagy axis in mediating TAX resistance in OC and demonstrated that pH/ROS-responsive nanomicelles carrying si-FDX1 effectively reversed this resistance both in vitro and in vivo. By precisely modulating copper homeostasis and autophagic flux, the approach enhanced chemotherapy sensitivity and offered a promising translational strategy for refractory OC. The strategy of co-delivering siRNA via nanomicelles offers unique advantages, including enhanced tumor targeting of the drug, reduced systemic side effects, and improved therapeutic efficacy. Given the challenges in OC treatment, this study demonstrates the potential of nanotechnology in overcoming drug resistance.

Despite these advances, several limitations remain. Although PPD/PDP@si-FDX1 showed strong therapeutic efficacy in vitro and in mouse models, its safety and efficacy in clinical applications require further validation. Moreover, the study primarily focused on copper-mediated regulation of the autophagy pathway in TAX resistance, while other mechanisms, such as genetic alterations and tumor microenvironmental factors, may also contribute and warrant deeper investigation. Future research will extend the evaluation of this nanomicellar platform to additional tumor models. Planned studies include (i) verifying the reproducibility of the FDX1–ULK1/ATG13–autophagy axis in OC organoid and patient-derived xenograft (PDX) models, (ii) reinforcing causal relationships through copper modulation and ULK1/ATG13 gene editing, and (iii) establishing a multi-parameter diagnostic framework while optimizing dosing regimens to assess clinical translatability. Further exploration of how PPD/PDP@si-FDX1 influences other cellular pathways and the immune microenvironment will clarify its broader mechanisms and expand its therapeutic scope. Upcoming preclinical studies will assess its long-term safety and efficacy across multiple cancer types to support potential clinical translation.

## Conclusion

Overall, the findings indicate that FDX1 markedly promotes TAX resistance in ovarian cancer cells by modulating intracellular copper homeostasis and activating the ULK1–ATG13 signaling axis. The pH/ROS-responsive nanomicelles developed in this study (PPD/PDP@si-FDX1) effectively reversed this resistance, enhancing the chemosensitivity of ovarian cancer cells and significantly improving the antitumor efficacy of TAX both in vitro and in vivo (Graphic abstract). These results highlight FDX1 as a potential therapeutic target and demonstrate the promise of nanotechnology-based siRNA delivery systems in overcoming drug resistance in OC.

## Supplementary Information

Supplementary Material 1.Figure S1. Differential expression of FDX1 in OC and validation of FDX1 silencing/overexpression effects. Note: (A) Analysis of FDX1 mRNA expression levels in tumor tissues (n=427) and normal tissues (n=88) based on TCGA and GTEx data; (B) Analysis of FDX1 protein expression levels in tumor tissues (n=100) and normal tissues (n=25) based on CPTAC data; (C) CCK-8 assay to assess cell viability in each group; (D-E) RT-qPCR (D) and Western Blot (E) analyses to determine FDX1 expression levels in parental and resistant cells; (F) Copper ion content in parental and resistant cells; (G) Western Blot analysis of LIAS and Lipoy-DLAT protein expression in parental and resistant cells across groups; (H) Western Blot screening to identify the most effective sequence for FDX1 silencing; (I) Western Blot validation of FDX1 overexpression effects. *p < 0.05,**p < 0.01, ***p< 0.001. Experiments were repeated three times.

Supplementary Material 2.Figure S2. Effects of FDX1 on copper levels, proliferation, migration, and invasion in SKOV3 and SKOV3-TR cells. Note: (A) Copper ion content in each cell group; (B) Western Blot analysis of LIAS and Lipoy-DLAT protein expression in each group; (C) Colony formation assay to evaluate cell proliferation in different groups; (D) Scratch assay to assess cell migration capacity, Scale bar = 200 μm; (E) Transwell assay to evaluate cell invasion capacity, Scale bar = 100 μm. *p < 0.05, **p < 0.01,***p < 0.001. Experiments were repeated three times.

Supplementary Material 3.Figure S3. Changes in autophagic flux in TAX-resistant OC cells identified through BafA1 treatment. Note: (A) Western Blot analysis of LC3B-I, LC3B-II, and P62 protein expression in each group; (B) Immunofluorescence staining for LC3-positive expression, Scale bar = 15 μm; (C) TEM images of mitochondrial morphology in each group, Scale bar = 1μm (left) and 500 nm (right), with red arrows indicating autophagosomes. *p< 0.05, **p < 0.01, ***p < 0.001. Experiments were repeated three times.

Supplementary Material 4.Figure S4. Effects of FDX1 on autophagy and TAX resistance in SKOV3 cells. Note: (A) Western Blot analysis of LC3B-I, LC3B-II, and P62 protein expression in each group; (B) JC-1 assay to measure MMP levels across groups; (C) Immunofluorescence staining for LC3-positive expression in each group; (D) TEM images showing mitochondrial morphology in each group, Scale bar = 1 μm (left) and 500 nm (right), with red arrows indicating autophagosomes; (E) CCK-8 assay to assess cell viability in DMSO and 3-MA treatment groups; (F) CCK-8 assay to evaluate cell viability in oe-FDX1 + DMSO and oe-FDX1 + 3-MA groups.*p < 0.05, **p< 0.01, ***p < 0.001. Experiments were repeated three times.

Supplementary Material 5.Figure S5. The roles of BL-918 and TTM in FDX1-mediated regulation of autophagy. Note: (A) Western blot analysis of A2780-TR cells detecting LC3-I/LC3-II and p62; grayscale quantification (normalized to Actin) is shown on the right. Treatment conditions: si-FDX1 cells treated with BL-918 or left untreated, with or without BafA1; (B) Same analyses as in (A) performed on SKOV3-TR cells; (C) Schematic of the cell experimental procedures; (D) Western Blot analysis of ULK1 and ATG13 protein expression in parental and resistant cells; (E) Western Blot analysis of ULK1 and ATG13 protein expression in A2780 and SKOV3 cells across groups. *p < 0.05,**p < 0.01, ***p< 0.001. Experiments were repeated three times.

Supplementary Material 6.Figure S6. Mechanistic investigation of FDX1's influence on autophagy and TAX resistance in SKOV3 cells. Note: (A) CCK-8 assay to evaluate cell viability across groups； (B) Western Blot analysis of ULK1, ATG13, LC3B-I, LC3B-II, and P62 protein expression across groups; (C) JC-1 assay to measure MMP levels in different groups; (D) Immunofluorescence staining for LC3-positive expression, Scale bar = 15 μm; (E) TEM images showing mitochondrial morphology, Scale bar = 1 μm (left) and 500 nm (right), with red arrows indicating autophagosomes; (F) Colony formation assay to assess cell proliferation across groups; (G) Scratch assay to measure cell migration capability, Scale bar = 100 μm; (H) Transwell assay to evaluate cell invasion capability, Scale bar = 50 μm. *p< 0.05, **p < 0.01, ***p < 0.001. Experiments were repeated three times.

Supplementary Material 7.Figure S7. Effects of PPD/PDP@si-FDX1 on OC cell proliferation, migration, and invasion. Note: (A) Colony formation assay to assess cell proliferation in different groups; (B) Scratch assay to evaluate cell migration capability, Scale bar = 100 μm; (C) Transwell assay to measure cell invasion capability, Scale bar = 50 μm. ***p< 0.001. Experiments were repeated three times.

Supplementary Material 8.Figure S8. H&E staining of mouse heart, spleen, liver, and kidney after 18 Days of treatment (scale bar = 100 μm).

Supplementary Material 9.Figure S9. Expression of key genes in mouse tumor tissues after 18 days of treatment. Note: (A) Immunofluorescence staining to detect FDX1-positive expression in tumor tissues, Scale bar = 50 μm; (B) Western Blot analysis of FDX1, ULK1, ATG13, LC3B-I, LC3B-II, and P62 protein expression in tumor tissues; (C) Quantitative analysis of data from panel (B); (D) Copper levels in tumor tissues across groups. *p< 0.05, **p < 0.01, ***p < 0.001. Each animal group consisted of six mice.

Supplementary Material 10. Table S1. siRNA sequence. Table S2. Primers for RT-qPCR analysis. Table S3. Western Blot antibodies information.

## Data Availability

All data generated or analyzed during this study are included in this article and/or its supplementary material files. Further enquiries can be directed to the corresponding author.

## Abbreviations

ANOVA: Analysis of Variance
ATG13: Autophagy Related 13
BCA: Bovine Serum Albumin
BP: Biological Processes
CCK-8: Cell Counting Kit-8
CC: Cellular Components
CLSM: Confocal Laser Scanning Microscope
CPTAC: Clinical Proteomic Tumor Analysis Consortium
DCC: Dicyclohexylcarbodiimide
DCM: Dichloromethane
DEP: Differentially Expressed Protein
DMMA: Dimethylmaleic Anhydride
DLS: Dynamic Light Scattering
FBS: Fetal Bovine Serum
FCM: Flow Cytometry
FDR: False Discovery Rate
FDX1: Ferredoxin 1
GTEx: Genotype-Tissue Expression
H&E: Hematoxylin and Eosin
HPLC: High-Performance Liquid Chromatography
IP: Immunoprecipitation
IT: Injection Time
MCS: Multicellular Spheroid
MMP: Mitochondrial Membrane Potential
MF: Molecular Functions
NHS: N-hydroxysuccinimide
OC: Ovarian Cancer
PEI: Polyethyleneimine
PDI: Polydispersity Index
PPD: PEG-PLL-DMMA
PPD/PDP@si-FDX1: pH/ROS-responsive Nanomicelles Loaded with si-FDX1
ROS: Reactive Oxygen Species
SD: Standard Deviation
siRNA: Small Interfering RNA
TAE: Tris-Acetate-EDTA
TAX: Paclitaxel
TCGA: The Cancer Genome Atlas
TEM: Transmission Electron Microscopy
THF: Tetrahydrofuran
ULK1: Unc-51 Like Autophagy Activating Kinase 1
