# Supplementary figures and images for: Ferrodoxin 1 (FDX1) drives paclitaxel resistance in ovarian cancer via copper metabolism and ULK1/ATG13-mediated autophagy: overcome by pH/ROS-responsive PPD/PDP@si-FDX1 nanomicelles
Source: J Exp Clin Cancer Res. 2026 Apr 23;45:104. doi: 10.1186/s13046-025-03589-z (PMC13104215; doi:10.1186/s13046-025-03589-z)

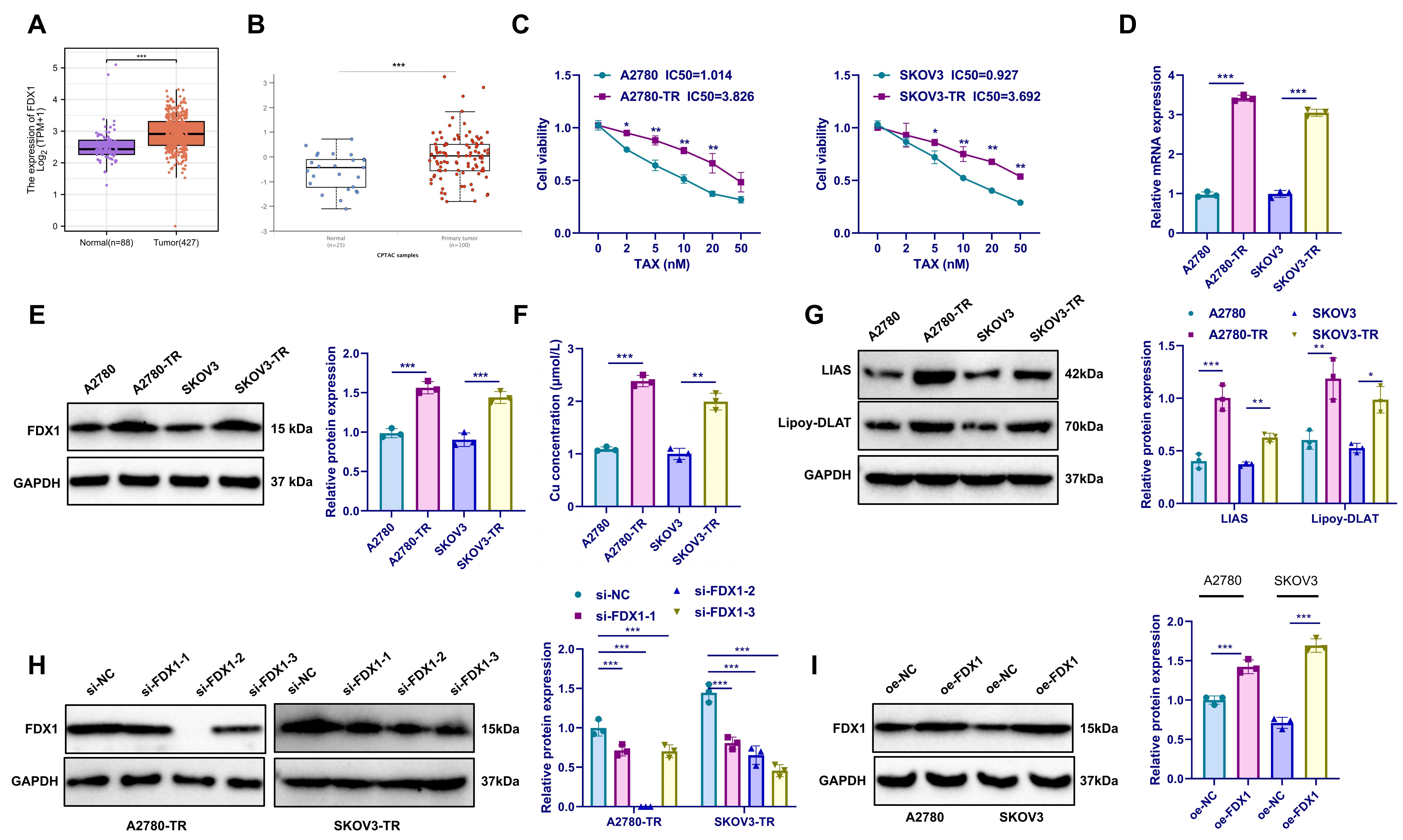

Supplement: Supplementary file 1 — Supplementary Material 1.Figure S1. Differential expression of FDX1 in OC and validation of FDX1 silencing/overexpression effects. Note: (A) Analysis of FDX1 mRNA expression levels in tumor tissues (n=427) and normal tissues (n=88) based on TCGA and GTEx data; (B) Analysis of FDX1 protein expression levels in tumor tissues (n=100) and normal tissues (n=25) based on CPTAC data; (C) CCK-8 assay to assess cell viability in each group; (D-E) RT-qPCR (D) and Western Blot (E) analyses to determine FDX1 expression levels in parental and resistant cells; (F) Copper ion content in parental and resistant cells; (G) Western Blot analysis of LIAS and Lipoy-DLAT protein expression in parental and resistant cells across groups; (H) Western Blot screening to identify the most effective sequence for FDX1 silencing; (I) Western Blot validation of FDX1 overexpression effects. *p < 0.05,**p < 0.01, ***p< 0.001. Experiments were repeated three times. [file 13046_2025_3589_MOESM1_ESM.jpg]

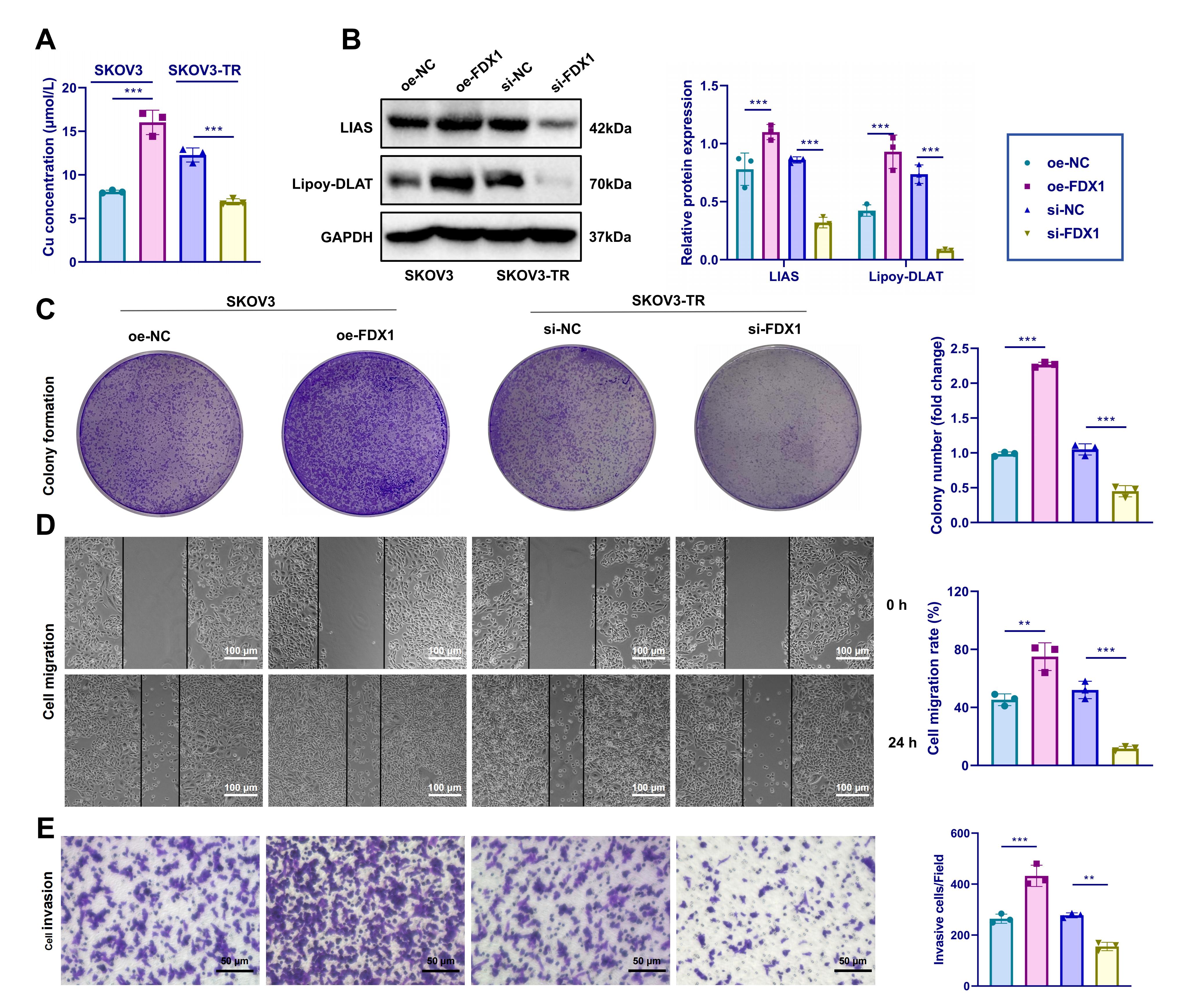

Supplement: Supplementary file 2 — Supplementary Material 2.Figure S2. Effects of FDX1 on copper levels, proliferation, migration, and invasion in SKOV3 and SKOV3-TR cells. Note: (A) Copper ion content in each cell group; (B) Western Blot analysis of LIAS and Lipoy-DLAT protein expression in each group; (C) Colony formation assay to evaluate cell proliferation in different groups; (D) Scratch assay to assess cell migration capacity, Scale bar = 200 μm; (E) Transwell assay to evaluate cell invasion capacity, Scale bar = 100 μm. *p < 0.05, **p < 0.01,***p < 0.001. Experiments were repeated three times. [file 13046_2025_3589_MOESM2_ESM.jpg]

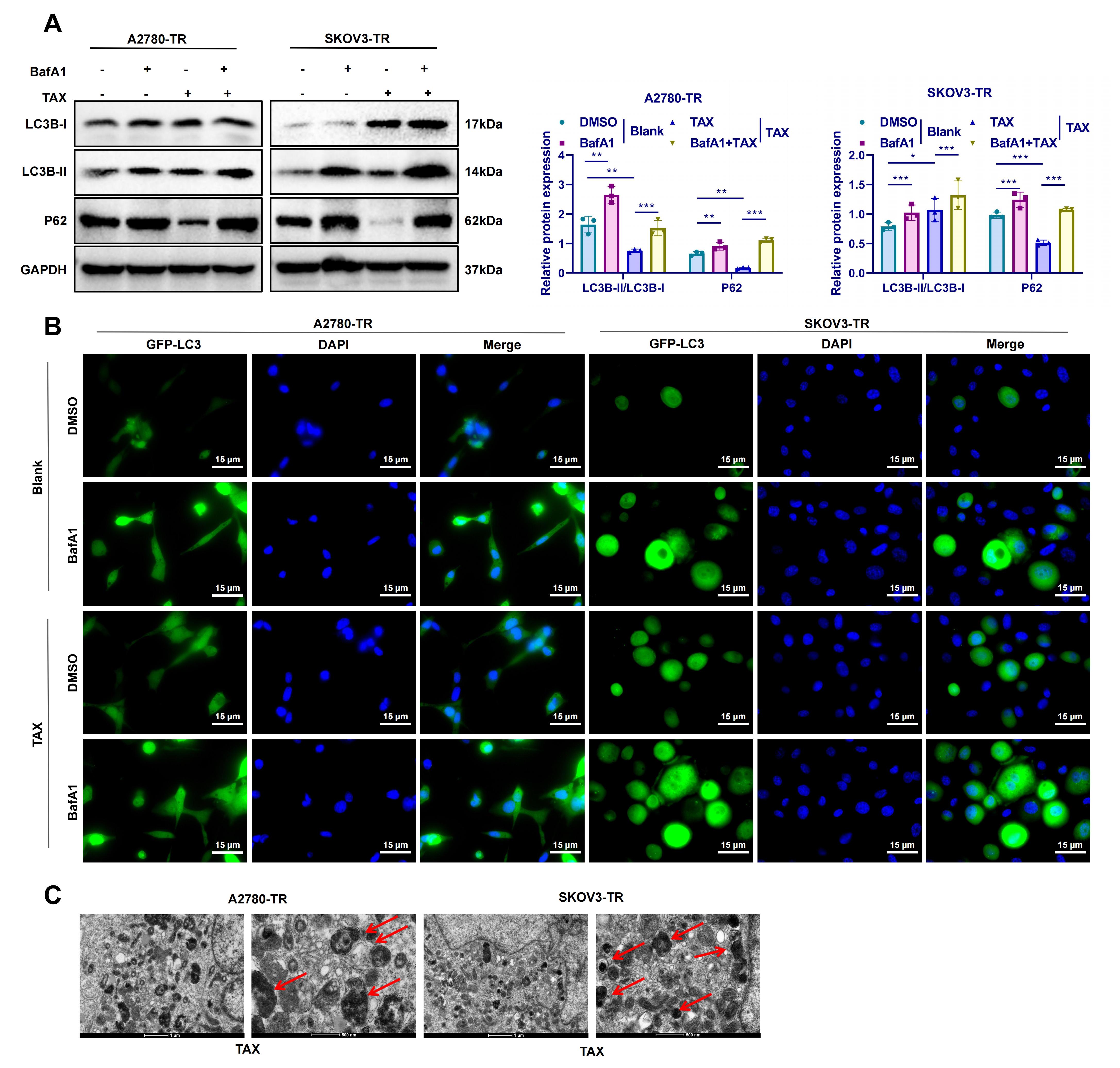

Supplement: Supplementary file 3 — Supplementary Material 3.Figure S3. Changes in autophagic flux in TAX-resistant OC cells identified through BafA1 treatment. Note: (A) Western Blot analysis of LC3B-I, LC3B-II, and P62 protein expression in each group; (B) Immunofluorescence staining for LC3-positive expression, Scale bar = 15 μm; (C) TEM images of mitochondrial morphology in each group, Scale bar = 1μm (left) and 500 nm (right), with red arrows indicating autophagosomes. *p< 0.05, **p < 0.01, ***p < 0.001. Experiments were repeated three times. [file 13046_2025_3589_MOESM3_ESM.jpg]

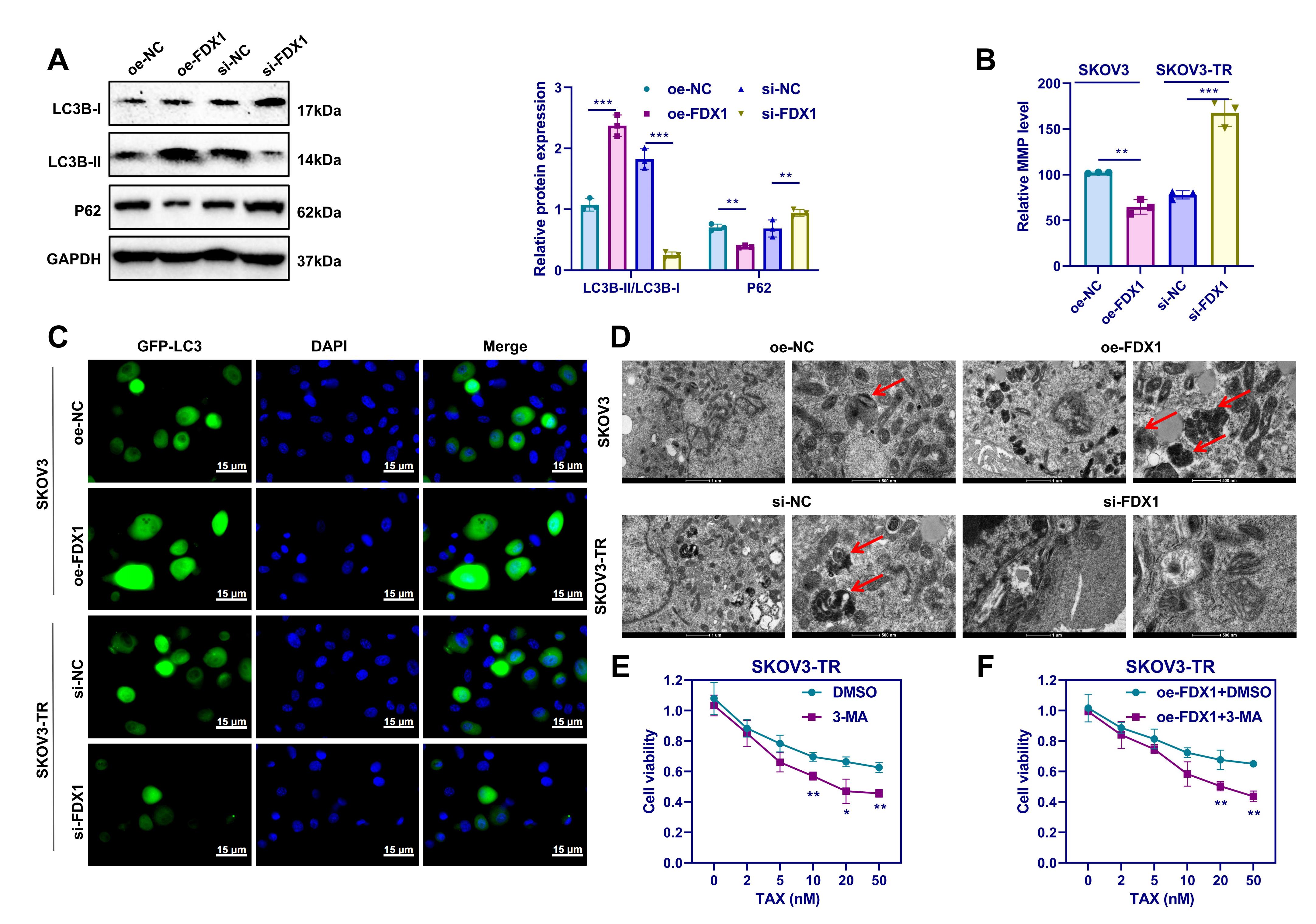

Supplement: Supplementary file 4 — Supplementary Material 4.Figure S4. Effects of FDX1 on autophagy and TAX resistance in SKOV3 cells. Note: (A) Western Blot analysis of LC3B-I, LC3B-II, and P62 protein expression in each group; (B) JC-1 assay to measure MMP levels across groups; (C) Immunofluorescence staining for LC3-positive expression in each group; (D) TEM images showing mitochondrial morphology in each group, Scale bar = 1 μm (left) and 500 nm (right), with red arrows indicating autophagosomes; (E) CCK-8 assay to assess cell viability in DMSO and 3-MA treatment groups; (F) CCK-8 assay to evaluate cell viability in oe-FDX1 + DMSO and oe-FDX1 + 3-MA groups.*p < 0.05, **p< 0.01, ***p < 0.001. Experiments were repeated three times. [file 13046_2025_3589_MOESM4_ESM.jpg]

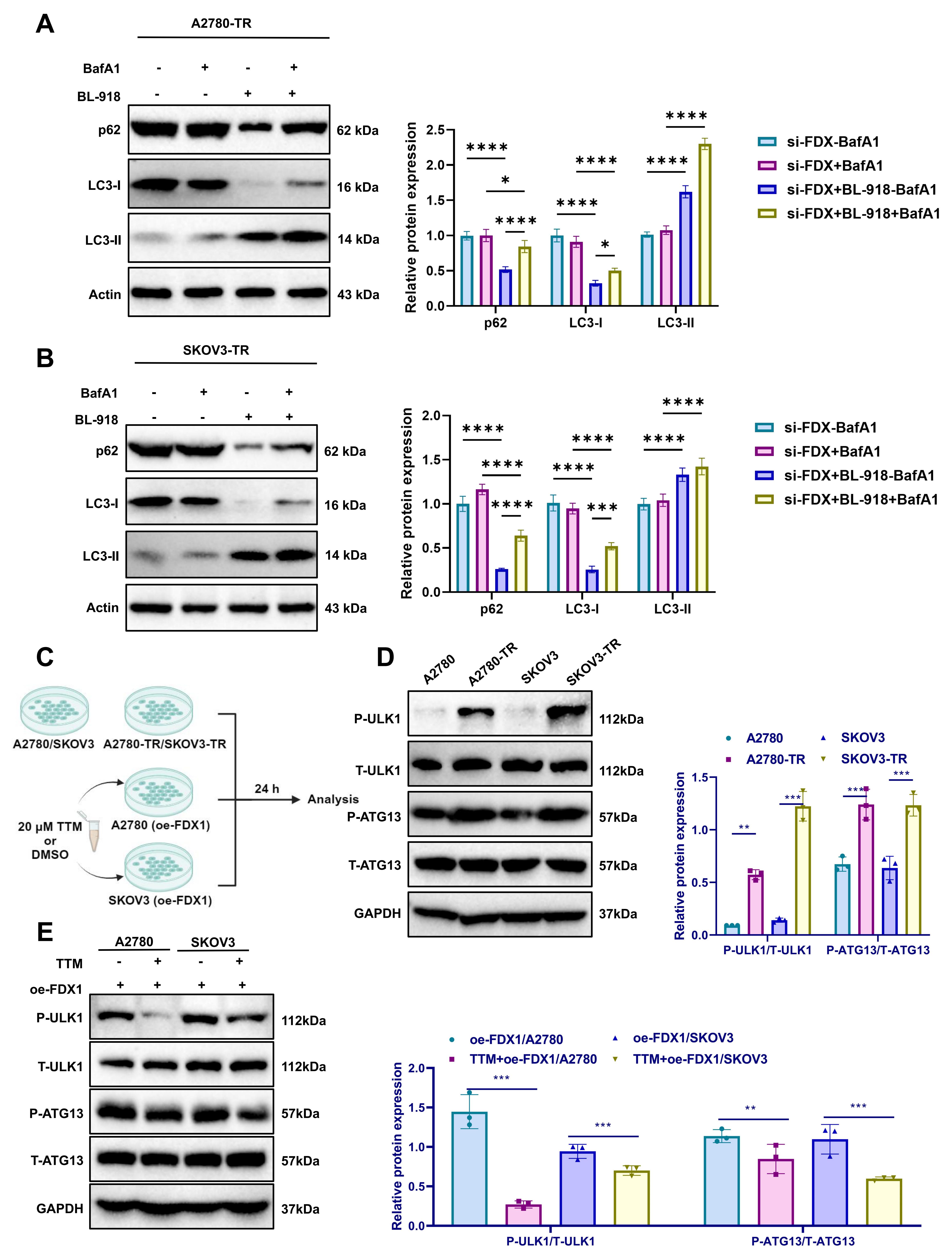

Supplement: Supplementary file 5 — Supplementary Material 5.Figure S5. The roles of BL-918 and TTM in FDX1-mediated regulation of autophagy. Note: (A) Western blot analysis of A2780-TR cells detecting LC3-I/LC3-II and p62; grayscale quantification (normalized to Actin) is shown on the right. Treatment conditions: si-FDX1 cells treated with BL-918 or left untreated, with or without BafA1; (B) Same analyses as in (A) performed on SKOV3-TR cells; (C) Schematic of the cell experimental procedures; (D) Western Blot analysis of ULK1 and ATG13 protein expression in parental and resistant cells; (E) Western Blot analysis of ULK1 and ATG13 protein expression in A2780 and SKOV3 cells across groups. *p < 0.05,**p < 0.01, ***p< 0.001. Experiments were repeated three times. [file 13046_2025_3589_MOESM5_ESM.jpg]

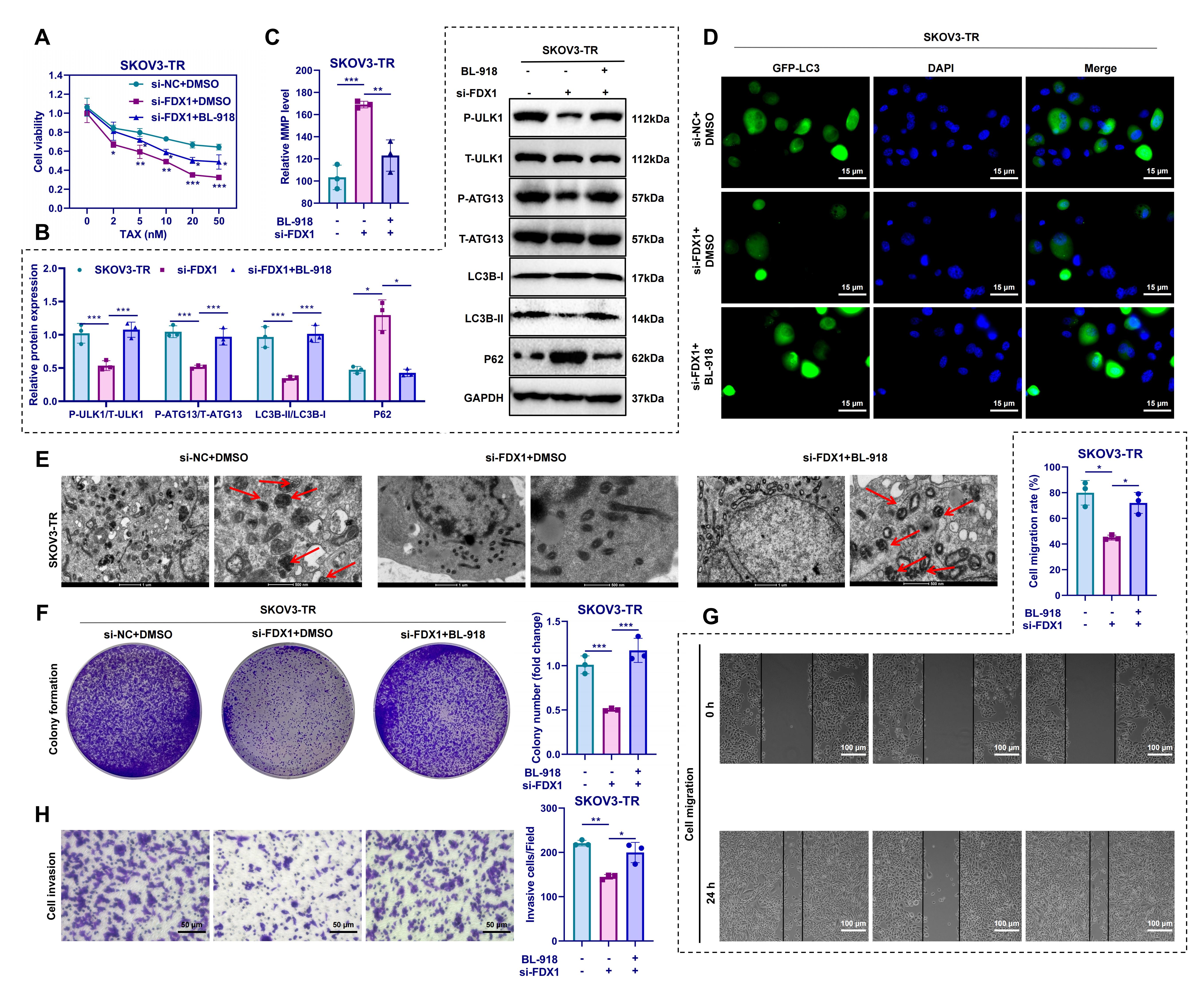

Supplement: Supplementary file 6 — Supplementary Material 6.Figure S6. Mechanistic investigation of FDX1's influence on autophagy and TAX resistance in SKOV3 cells. Note: (A) CCK-8 assay to evaluate cell viability across groups； (B) Western Blot analysis of ULK1, ATG13, LC3B-I, LC3B-II, and P62 protein expression across groups; (C) JC-1 assay to measure MMP levels in different groups; (D) Immunofluorescence staining for LC3-positive expression, Scale bar = 15 μm; (E) TEM images showing mitochondrial morphology, Scale bar = 1 μm (left) and 500 nm (right), with red arrows indicating autophagosomes; (F) Colony formation assay to assess cell proliferation across groups; (G) Scratch assay to measure cell migration capability, Scale bar = 100 μm; (H) Transwell assay to evaluate cell invasion capability, Scale bar = 50 μm. *p< 0.05, **p < 0.01, ***p < 0.001. Experiments were repeated three times. [file 13046_2025_3589_MOESM6_ESM.jpg]

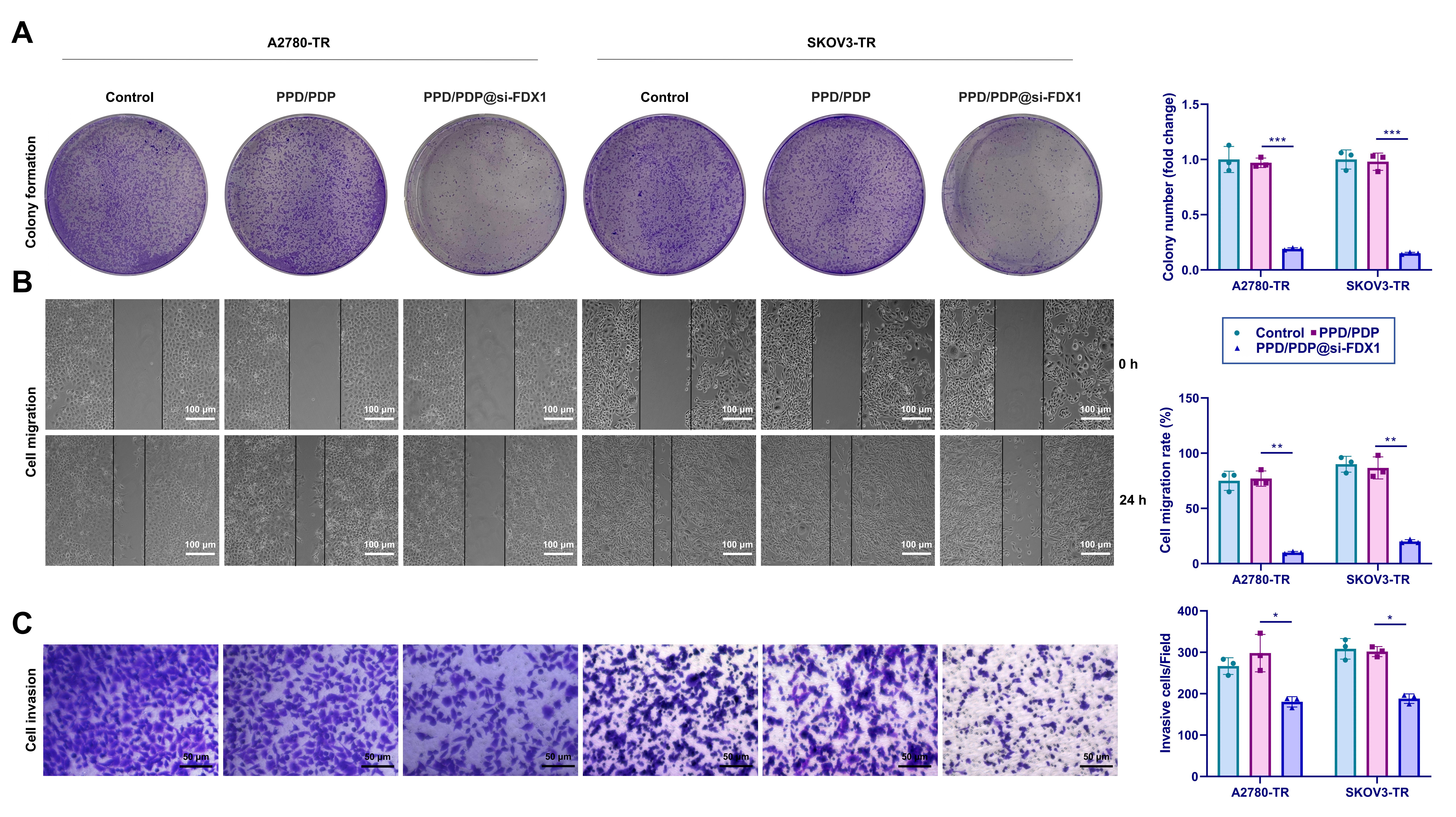

Supplement: Supplementary file 7 — Supplementary Material 7.Figure S7. Effects of PPD/PDP@si-FDX1 on OC cell proliferation, migration, and invasion. Note: (A) Colony formation assay to assess cell proliferation in different groups; (B) Scratch assay to evaluate cell migration capability, Scale bar = 100 μm; (C) Transwell assay to measure cell invasion capability, Scale bar = 50 μm. ***p< 0.001. Experiments were repeated three times. [file 13046_2025_3589_MOESM7_ESM.jpg]

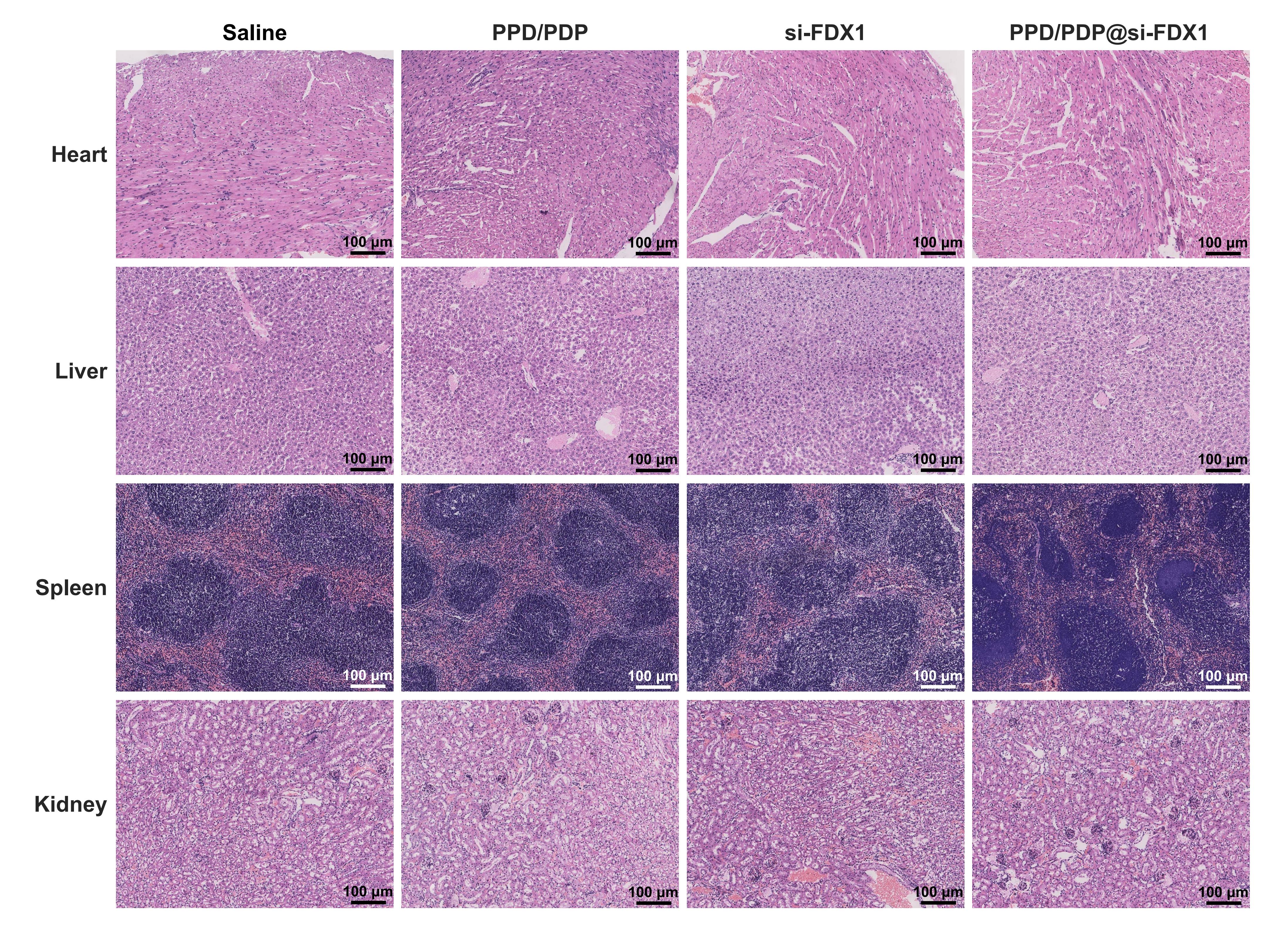

Supplement: Supplementary file 8 — Supplementary Material 8.Figure S8. H&E staining of mouse heart, spleen, liver, and kidney after 18 Days of treatment (scale bar = 100 μm). [file 13046_2025_3589_MOESM8_ESM.jpg]

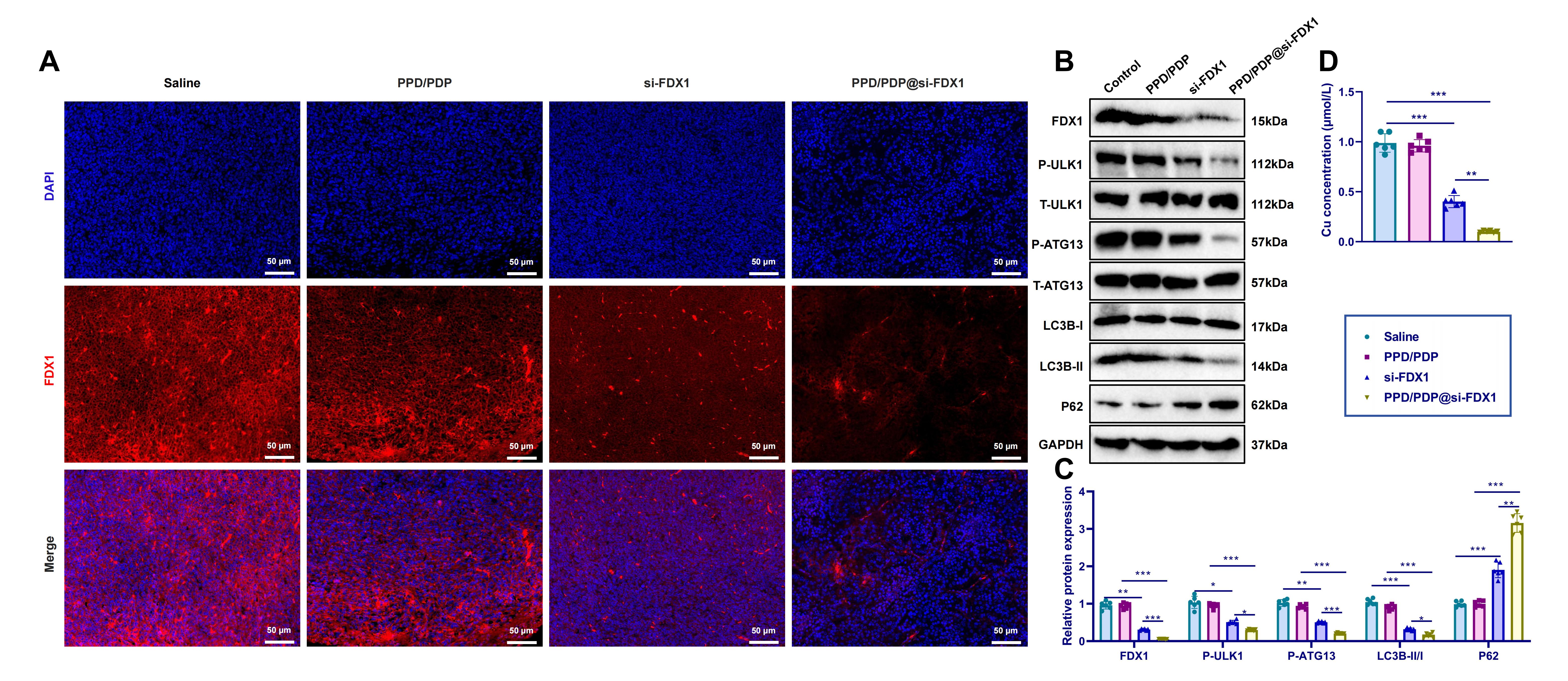

Supplement: Supplementary file 9 — Supplementary Material 9.Figure S9. Expression of key genes in mouse tumor tissues after 18 days of treatment. Note: (A) Immunofluorescence staining to detect FDX1-positive expression in tumor tissues, Scale bar = 50 μm; (B) Western Blot analysis of FDX1, ULK1, ATG13, LC3B-I, LC3B-II, and P62 protein expression in tumor tissues; (C) Quantitative analysis of data from panel (B); (D) Copper levels in tumor tissues across groups. *p< 0.05, **p < 0.01, ***p < 0.001. Each animal group consisted of six mice. [file 13046_2025_3589_MOESM9_ESM.jpg]
