# Supplementary material for: Ferrodoxin 1 (FDX1) drives paclitaxel resistance in ovarian cancer via copper metabolism and ULK1/ATG13-mediated autophagy: overcome by pH/ROS-responsive PPD/PDP@si-FDX1 nanomicelles
Source: J Exp Clin Cancer Res. 2026 Apr 23;45:104. doi: 10.1186/s13046-025-03589-z (PMC13104215; doi:10.1186/s13046-025-03589-z)
Supplement: Supplementary file 10 — Supplementary Material 10. Table S1. siRNA sequence. Table S2. Primers for RT-qPCR analysis. Table S3. Western Blot antibodies information. [file 13046_2025_3589_MOESM10_ESM.docx]

**Table S1. siRNA sequence.**

| **Name** | **Sequence(5'-3')** |
| --- | --- |
| si-FDX1-1 | TCCACTTTATAAACCGTGATGGTGA |
| si-FDX1-2 | CCACTTTATAAACCGTGATGGTGAA |
| si-FDX1-3 | TATAAACCGTGATGGTGAAACATTA |

**Table S2. Primers for RT-qPCR analysis**

| Gene name | Forward primer sequence(5’-3’) | Reverse primer sequencee(5’-3’) |
| --- | --- | --- |
| *Gapdh* | GTGGACCTGACCTGCCGTCTAG | GAGTGGGTGTCGCTGTTGAAGTC |
| *FDX1* | TTCAACCTGTCACCTCATCTTTG | TGCCAGATCGAGCATGTCATT |

**Table S3. Western Blot antibodies information.**

| **Target Name** | **Manufacturer** | **Catalog Number** | **Dilution Ratio** |
| --- | --- | --- | --- |
| FDX1 | Abcam | ab108257 | 1: 2000 |
| LIAS | Abcam | ab96302 | 1: 500 |
| DLAT | Abcam | ab172617 | 1: 1000 |
| LC3B-I | Abcam | ab52628 | 1: 50000 |
| LC3B-II | Abcam | ab192890 | 1: 2000 |
| P62 | Abcam | ab109012 | 1: 10000 |
| P-ULK1 | Abcam | ab203207 | 1: 100 |
| T-ULK1 | Abcam | ab167139 | 1: 1000 |
| P-ATG13 | Abnova | PAB19948 | 1: 1000 |
| T-ATG13 | Abcam | ab105392 | 1: 1000 |
| GAPDH | Abcam | ab9485 | 1: 2500 |

Note: Abcam, UK; Abnova, USA.
